# UPLC-ESI-TOF MS Profiling Discriminates Biomarkers in Authentic and Adulterated Italian Samples of Saffron (*Crocus sativus* L.)

**DOI:** 10.1021/acsfoodscitech.4c00340

**Published:** 2024-07-04

**Authors:** Lucrezia Angeli, Ksenia Morozova, Corinna Dawid, Matteo Scampicchio, Timo D. Stark

**Affiliations:** † Faculty for Agricultural, Environmental, and Food Sciences, Free University of Bozen-Bolzano, 39100 Bolzano, Italy; ‡ Professorship for Functional Phytometabolomics, TUM School of Life Sciences, Technical University of Munich, Lise-Meitner-Str. 34, 85354 Freising, Germany; § Food Chemistry and Molecular Sensory Science, Technical University of Munich, Lise-Meitner-Str. 34, 85354 Freising, Germany

**Keywords:** profiling, adulteration, high-resolution mass spectrometry, crocins, PCA

## Abstract

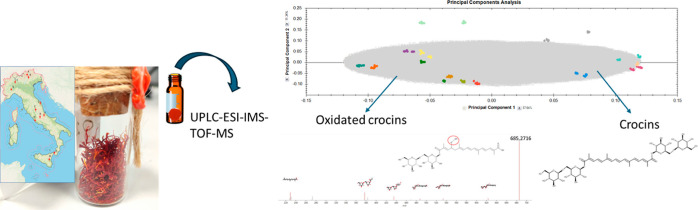

Italian saffron (*Crocus sativus* L.) is gaining visibility due to its high quality and difference in growing area. In this study, the metabolite composition and quality of Italian saffron samples purchased from local producers and supermarkets were investigated using an untargeted metabolomics approach using UPLC-ESI-TOF MS with simultaneous acquisition of low- and high-collision energy mass spectrometry (MS^e^). Unsupervised statistical method (PCA) highlighted significant differences in the metabolomes, even if not related to the geographical origin. OPLS-DA revealed 9(*S*)-,10-(*S*)-,13-(*S*)-tri-hydroxy-11-(*E*)-octadecenoic acid as the most decisive compound to distinguish supermarket saffron, while oxidized crocins represented the most valuable markers to further describe the quality of saffron, even in locally produced samples. Known adulterations with paprika and turmeric were detected at a limit of 10%, and the increasing signals of cyclocurcumin was a significant biomarker for turmeric contamination. The results were underlined with conventional and kinetic antioxidant assays.

## 1 Introduction

Saffron (*Crocus sativus* L.) is a perennial herbaceous geophyte in the Iridaceae family.^1^ The dried stigmas are used as a common spice to improve color, taste, and aroma to foods in Middle Eastern and Spanish cuisine.^2^ The main chemical coloring compounds in saffron are crocins, which are water-soluble carotenoids containing crocetin and a sugar moiety, mainly gentiobiose. Broad research into crocins has suggested that their biological effect is related to the strong free-radical scavenging activity and antioxidant properties.^3,4^

Saffron is only cultivated and produced in several countries, including Afghanistan, Azerbaijan, China, Iran, India, Greece, Morocco, and Spain. Among which Iran is the biggest producer with a market share of 90% of the produced saffron globally.^4^ Recently, there has been an increasing interest in saffron grown at different latitudes, including in Italy. In Italy, saffron is mainly cultivated near L’Aquila (Piana di Navelli), in the regions of Sardinia (Province of Medio Campidano), Tuscany (San Gimignano, Florence Hills, and Maremma), and Umbria (Cascia and Città della Pieve).^5^ However, due to climate change, saffron is nowadays also grown at higher altitudes, such as in the regions of Piemonte, Lombardy, and Trentino-Alto Adige. The increasing interest in *C. sativus* cultivation in the Italian Alps has led to expanded production of this spice in recent years. Thus, some studies have focused on the quality assessment of saffron obtained from the alpine environment.^1,6^ However, to the best of our knowledge, no studies so far have compared the composition of saffron coming from Northern Italy with saffron from other Italian regions.

Determining the quality of saffron is extremely important. Indeed, low yield, manual harvesting, and the drying process are responsible for the high price of saffron, considered the most expensive spice in the world and, for this reason, among the most frequently adulterated food products.^7^ The normative ISO3632 is used to assess saffron quality through the spectrophotometric and chromatographic quantification of picrocrocin, safranal, and crocins.^8^ However, when saffron is adulterated with other plant species, such as turmeric, safflower, calendula, etc., this protocol fails at determining saffron quality at levels below 20%.^9^ Consequently, there is a growing need for the continuous improvement of sensitive analytical techniques to assess saffron quality.^4^ Therefore, the identification of biomarkers for the discrimination of saffron according to its authenticity, origins, and quality remains a widely discussed subject.

Several studies highlight the suitability of different analysis methods for this purpose, such as liquid chromatography coupled with a diode array detector (UPLC-DAD), mass spectrometry (MS), or, more recently, nuclear magnetic resonance (NMR) spectroscopy.^2,10,18^ The untargeted ultraperformance liquid chromatography-electrospray ionization-time-of-flight mass spectrometry (UPLC-ESI-TOF MS) fast screening method and chemometrics analysis described in the present study were used to investigate the quality of saffron samples cultivated in different geographic areas in Italy and their adulteration with turmeric and paprika powders. Moreover, the antioxidant properties of these extracts were investigated to assess their quality. Therefore, the aim of this research was to (i) identify potential biomarkers for the determination of saffron quality, (ii) detect adulteration limits below 15%, and (iii) evaluate the antioxidant properties of the extracts with spectrophotometric and kinetic approaches as indicator of quality.

## 2 Materials and Methods

### 2.1 Chemicals and Reagents

Methanol, acetonitrile, and formic acid MS grade were purchased from Fisher Scientific (Schwerte, Germany), and crocetin-digentiobioseester (α-crocin), crocetin-gentiobiosylglucosylester, *trans*-crocetin-gentiobiosylester, 2-*O*-α-d-glucopyranosyl-l-ascorbic acid, and (9*S*,10*S*,13*S*)-trihydroxy-11-(*E*)-octadecenoic acid with purity higher than 98% were purchased from Sigma-Aldrich (Steinheim, Germany). A Milli-Q Reference A+ water purification system was used to prepare water for all experiments (Millipore, Schwalbach, Germany).

### 2.2 Plant Material

Saffron samples harvested in 2022 were purchased from Italian local producers and supermarkets, according to Table 1. Two different samples were purchased for each company and were treated as biological replicates. Paprika powder and turmeric powder were purchased from a local supermarket.

**Table 1:** Sample Name, Company Location, Type of Material, and Year of Harvesting of Saffron Samples

sample name	company location	type of material	year
A	South Tyrol (BZ)	dried stigmas	2022
B	Trentino (TN)	dried stigmas	2022
C	Lombardia (CO)	dried stigmas	2022
D	Calabria (RC)	dried stigmas	2022
E	Umbria (PG)	dried stigmas	2022
F	Abruzzo (CH)	dried stigmas	2022
G	Piemonte (AT)	dried stigmas	2022
H	Lazio (RO)	dried stigmas	2022
I	“Primia” (supermarket)	powder	unknown
J	“Tre cuochi” (supermarket)	powder	unknown
K	“Kotany” (supermarket)	dried stigmas	unknown
L	Marche (AN)	dried stigmas	2022
M	Sicilia (CT)	dried stigmas	2022
N	Abruzzo (AQ)	dried stigmas	2022

### 2.3 Extraction of Secondary Metabolites from the Plant Material

First, the saffron samples were ground with a Precellys Evolution (VWR, Darmstadt, Germany) at a speed of 8000 rpm, with 3 cycles of 25 s and intervals of 20 s. Then, the obtained powders were freeze-dried and extracted with different ratios (10, 50, 90%, v/v) of aqueous methanol and aqueous acetonitrile to optimize the extraction method. The 5 mg/mL solutions were extracted with the Precellys evolution (VWR) at a speed of 628.3 rad/s, with 3 cycles of 25 s and intervals of 20 s, centrifuged at 418.7 rad/s for 10 min at 20 °C, and the supernatant was filtered with 0.45 μm membrane filters (VWR) and stored at −30 °C prior to further analysis.

### 2.4 UPLC-TOF-MS for Untargeted Analysis

A Vion HDMS ESI-TWIMS-ToF-MS instrument (Waters, Manchester, U.K.) was used to record high-resolution mass spectra. For chromatographic separation, an Acquity i-class UPLC core system (Waters, Milford, MA, USA) which consisted of a binary solvent manager, sample manager, and column oven was connected to the mass spectrometer. To obtain untargeted data, the saffron samples (3 μL each, 3 injections/sample) were subjected to UPLC-TOF-MS profiling using a BEH C18 column (150 × 2.1 mm, 1.7 μm, Waters, Manchester, UK). Chromatographic separation was performed at a flow rate of 0.4 mL/min at 50 °C with a gradient consisting of aqueous formic acid (0.1%) as eluent A and acetonitrile (0.1% formic acid) as eluent B. The gradient started with 5% B, which remained constant for 1 min, followed by an increase to 20% B within 1 min. The content of B was then increased to 30% B within 2 min, then to 50% after 2 min, and to 99% after 2 min. 99% B was kept for 0.9 min and dropped to 5% after 0.5 min, and it was held for 1. The run time was 10 min. TOF-MS analyses were carried out in negative and positive electrospray ionization modes using the following ion source parameters: capillary voltage of 1.5 kV in both positive and negative modes; sampling cone, 30 V; source offset, 80 V; source temperature, 150 °C; desolvation temperature, 450 °C; cone gas flow, 50 L/h; and desolvation gas, 850 L/h. The detector voltage was set at 2700 V. Data processing was performed by using UNIFI 1.8 (Waters, Manchester, UK) and the elemental composition tool for determining the accurate mass. All data were lock-mass-corrected on the pentapeptide leucine enkephaline (Tyr-Gly-Gly-Phe-Leu) in a solution (0.1 ng/μL) of MeCN/0.1% HCO_2_H (1:1, v/v). Scan time for the lock mass was set to 0.3 s, at intervals of 15 and 3 scans to average with a mass window of ±0.3 Da. Calibration of the Vion in the range from *m*/*z* 50 to 1200 was performed using a solution of MajorMix (Waters). The UPLC and Vion systems were operated with the UNIFI software (Waters). Collision energy ramp for HDMS^e^ was set from 20 to 40 eV. Further details of the Vion IMS qTof instrument are listed in the SI (Table S1).

### 2.5 Adulteration with Paprika and Turmeric

The quality control (QC) generated by an aliquot from all saffron extracts, sample A (from North Italy), sample N (from Central Italy), and sample M (from South Italy), was spiked with 10, 25, and 50% of paprika (*Capsicum annuum* L.) or turmeric extract (*Curcuma longa*), respectively. Paprika and turmeric were extracted with 90% aqueous CH_3_OH as described for the saffron extraction.

### 2.6 Antioxidant Activity with the DPPH^•^ Kinetic Approach

The kinetic-based DPPH^•^ method was performed with a stopped-flow system (RX2000, Applied Photophysics, Leatherhead, UK) equipped with a pneumatic pump, a quartz flow cell, and a Cary 60 UV–VIS spectrophotometer (Agilent Technology, Santa Clara, CA, USA). The method of Angeli et al. with slight modifications was used.^11^ Briefly, the two syringes were loaded with 200 μM DPPH^•^ solution, and saffron extract was standardized at 60 μM gallic acid equivalents (GAE). Priming was performed before every run by flushing the two reagents into the system. As soon as the pneumatic drive was pressed, equal volumes of the two solutions were mixed and transferred into the quartz flow cell, with a max delay of 6 ms. The resulting absorbance of the reaction mixture was recorded every 18 ms, at a wavelength of 515 nm. The concentration of DPPH^•^ was calculated from the recorded absorbance signal by applying the Beer–Lambert law. At this purpose, the molar extinction coefficient of DPPH^•^ in methanol (ε_515_) was determined from the absorbance of increasing standard solutions, leading to values of ε_515_ equal to 11,200 ± 400 M^–1^ cm^–1^, in agreement with that found elsewhere.^12^ Simulation and fitting of the reaction kinetic data were performed with Copasi (version 4.29). Simulated DPPH^•^ consumption was obtained from solving a set of differential equations derived by the law of mass action applied to eqs 1 and 2.
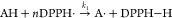
1
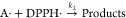
2

Optimal values of the kinetic parameters (*k*_1_ and *k*_2_) and the reaction stoichiometry (*n*) were obtained by minimizing, through iteration, the sum of squared errors between the experimental and simulated data. Each experimental point reported in this article is the average of three independent replicates.

### 2.7 Total Crocin Content

Total crocins were measured with the method of Zhang et al. with slight modifications.^3^ Briefly, a calibration curve for α-crocin was obtained (*y* = 6.14*x* + 0.01, linear from 0 to 0.4 mg/mL, *r*^2^ = 0.999), and the absorbance of standard and extracts was recorded at 440 nm with an Infinite M Nano + spectrophotometer (Tecan, Mannedorf, Switzerland). The concentration of crocins in the extracts was reported as milligrams mL^–1^ and adjusted for the dilution factor.

### 2.8 Total Phenolic Content

Total phenolic content (TPC) was estimated with the Folin–Ciocalteu method with slight modifications.^13^ Briefly, a volume of the saffron sample (130 μL) was mixed with distilled water (1 mL) and Folin reagent (130 μL). After 5 min, Na_2_CO_3_ solution (130 μL, 20%) was added. The mixture was vortexed, incubated for 2 h in the dark at 25 °C, and transferred in a microplate well (UV-StarR microplate, 96 wells, Greiner Bio one, Frickenhausen, Germany). The absorbance was measured at 765 nm with a spectrophotometer (Infinite M Nano+, Tecan, Mannedorf, Switzerland). Results were expressed as mg mL^–1^ of GAE from a calibration curve built with standard solutions of gallic acid (*y* = 4.51*x* + 0.03, linear from 0 to 0.8 mg/mL, *r*^2^ = 0.998).

### 2.9 Statistical Analysis

Processing of MS raw data was done with Progenesis QI, using the following peak picking conditions: all runs, limits automatic, sensitivity 3, and retention time limits 0.5–9.25 min. Alignment was performed using a QC injection as a reference. Compounds used for principal component analysis (PCA) were filtered by means of ANOVA *p* value ≤0.05 and a fold change of ≥2. The processed data were exported to EZinfo version 3.0 (Umetrics AB, Umeå, Sweden), where the matrix was analyzed by PCA with pareto scaling.^14^ Group differences were calculated using orthogonal partial least-squares discriminant analysis (OPLS-DA) highlighted as S-plots.^15^ The R2 and Q2 values for all of the S-plots calculated are between at least 97 and 99%. The annotation of MS compounds was based on the fragmentation spectra of reference compounds, the match with existing libraries and predicted compounds, and the published literature. For compound proposals, an error less than 5 ppm was used.

Statistical analysis of kinetic data was performed with Copasi software (version 4.29). Basic statistics, such as mean and standard deviation, were obtained by Microsoft Excel (Version 2211 Build 16.0.15831.20098).

## 3 Results and Discussion

### 3.1 Optimization of Extraction and Separation

Preliminary experiments explored various solvent ratios to optimize saffron extraction efficiency. 10, 50, and 90% (v/v) aqueous acetonitrile or aqueous methanol were used to determine the highest peak intensities and the best resolution. Methanol significantly outperformed acetonitrile in terms of peak shape, giving narrower peaks, indicating better resolution. However, the comparison showed no differences in the total number of peaks detectable, suggesting a similar ability to extract compounds (data not shown). Therefore, 90% aqueous methanol (v/v) was selected to achieve superior solvation properties for a greater number of compounds, especially the crocetin esters, polar compounds requiring a polar solvent.^16^ Also, the separation conditions were improved by modifying the chromatographic gradient.

### 3.2 Untargeted Metabolomics to Select Markers of Different Commercial Saffron Samples

**Figure 1:**
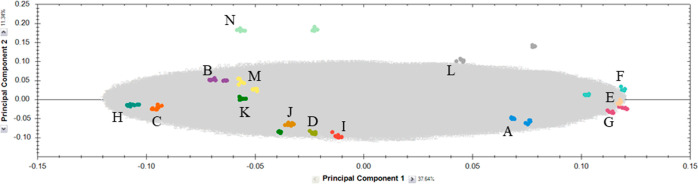
Score plot (comp. 1 vs comp. 2) of UPLC-ESI-TOF MS full scan analysis (50–1200 Da, ESI^–^, high-resolution mode) of saffron samples (three technical replicates, two extraction replicates, and two biological replicates per samples). Letters A-N refer to Table 1.

An untargeted metabolomic approach was used to assess the metabolite diversity between supermarket saffron samples and those obtained from local Italian producers. This strategy consisted of using an UPLC-ESI-TOF MS, with simultaneous acquisition of low and high collision energy (MS^e^). The analysis followed a Progenesis QI workflow, including steps from data importation, alignment review, experiment design setup, peak picking, deconvolution review, compounds review, compound statistics, and statistical analysis. This comprehensive procedure allowed the comparison of metabolite profiles, highlighting any differences between the authentic Italian samples (from local producers) and from those purchased in supermarkets. Principal component analysis (PCA), supported by ANOVA (*p*-value ≤0.05 and fold change ≥2), facilitated the discrimination of authentic Italian saffron samples from those purchased in supermarkets, as visually presented in Figure 1. This figure shows the separation of the saffron samples in terms of PC1, which explained 37% of the total variance, and PC2, which explained 11% of the total variance.

In our PCA, most saffron samples showed robust clustering according to their biological and technical replicates, underlining the reproducibility of the UPLC-ESI-TOF MS method. Among others, samples N (Abruzzo, AQ), L (Marche, AN), and K (“Kotany” supermarket) were exceptions, suggesting unique metabolite profiles, possibly due to specific local conditions or processing differences. Contrary to initial expectations, PCA did not reveal any clear clusters based on geographical origin. This observation suggests that the secondary metabolite composition of saffron is influenced by a complex interplay of factors, such as soil composition, climate, and postharvest processing, rather than geography alone.^17^

Interestingly, supermarket samples (I, J, and K) formed a distinct cluster together with sample D, which was separated from most local samples. This separation suggests differences in metabolite profiles, possibly due to processing or storage differences between supermarket and local saffron. Two main groups emerged along PC1: group X, consisting of samples from different Italian regions (L, A, F, E, and G), and group Y, comprising the remaining samples. This division suggests that saffron samples, although not geographically segregated, can be differentiated on the basis of subtle metabolomic variations.

**Figure 2:**
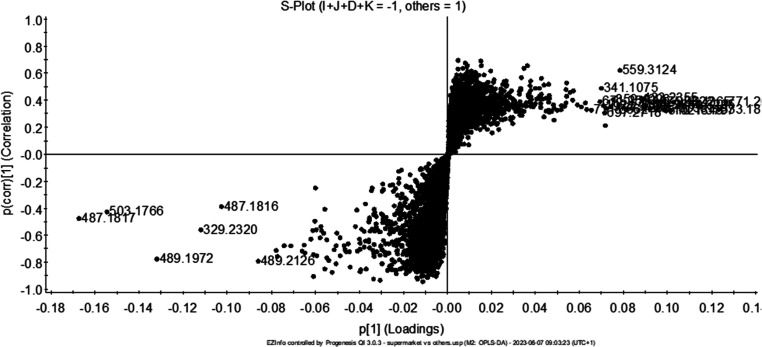
S-Plot of supermarket samples (I, J, K) and D (–1) versus all the others (A, B, C, E, F, G, H, L, M, N; +1).

The comparison between supermarket samples against all others was performed using orthogonal partial least-squares discriminant analysis (OPLS-DA). This statistical analysis allowed us to highlight and compare the specific characteristics of the supermarket samples, which might be considered of lower quality due to the lower price, with the local samples to discover distinctive metabolites. To aid this comparison and visual interpretation of the data, we used S-plots analysis. This is a graphical tool to identify significant metabolites contributing to differences between groups. S-plots are particularly useful for highlighting compounds that not only significantly discriminate between sample groups but are also measured with high reliability. On the plot shown in Figure 2, the horizontal axis represents the magnitude of each metabolite contribution to discriminating between groups, while the vertical axis indicates the confidence in these contributions. Metabolites that appear far from the origin along the *x*-axis of this plot are considered to be highly influential, while those that appear far from the origin along the *y*-axis are considered reliable markers of differentiation.

Using an S-plot, we identified several metabolites with pronounced differences between supermarket and local samples. For example, metabolites at *m*/*z* 503.1766, 489.1972, 487.1817, and 329.2320 eluted, respectively, at a RT of 3,34, 3.37, 3.86, and 6.55 min, emerged as the key contributors to the distinct profile of the supermarket samples.

In particular, the compound at *m*/*z* 503.1766, with elemental composition of C_22_H_32_O_13_, was proposed to be 5-hydroxy-7,7-dimethyl-4,5,6,7-tetrahydro-3H-isobenzo-furanone-5-*O*-β-d-gentiobioside (**1**), a known saffron compound.^2^

Particularly intriguing was identifying a novel compound not previously reported in the literature when analyzing saffron. This compound corresponded to the ion at *m*/*z* = 489.1972. Given its fragmentation pattern, similarity in RT to (**1**), and with assumed elemental composition of C_22_H_34_O_12_, this compound was proposed to be 5-((3,4-dihydroxy-6-(((3,4,6-trihydroxy-5-(hydroxymethyl)oxan-2-yl)oxy)methyl)oxan-2-yl)oxy)-7,7-dimethyl-4,5,6,7-tetrahydro-2-benzofuran-1(3*H*)-one (**2**), which differed from **1** for a hydroxyl group and the saturation of a double bond, corresponding to the loss of an oxygen atom and the gain of 2H. This compound was never described before in saffron.

A further compound that has never been reported for saffron before was the ion at *m*/*z* = 487.1817. Based on its fragmentation patterns, and calculated elemental composition of C_22_H_32_O_12_, this ion was proposed as 5-((3,4-dihydroxy-6-(((3,4,6-trihydroxy-5-(hydroxymethyl)oxan-2-yl)oxy)methyl)oxan-2-yl)oxy)-7,7-dimethyl-4,7-dihydro-2-benzofuran-1(3*H*)-one (**3**). Compared to **2**, the fragmentation pattern and the precursor ion of **3** led to the formation of a double bond corresponding to the loss of 2H.

Finally, the ion at *m*/*z* 329.2320 was unequivocally identified as (9*S*,10*S*,13*S*)-trihydroxy-(11*E*)-octadecenoic acid (**4**) by using injection of the corresponding analytical standard as well as spiking (Figure S1). Trihydroxy fatty acids are degradation products of fatty acids that were previously described as markers to distinguish commercial saffron samples from locally cultivated ones, supporting our findings.^18^ Overall, the results illustrated in Figure 1 and 2, and summarized in Table 2, highlight the metabolomic variance between supermarket and locally sourced saffron. These findings, which also include the discovery of new metabolites never reported in saffron, open new possibilities for the authentication of saffron samples and could provide an alternative approach to discriminate between commercially processed and traditionally cultivated saffron samples based on their metabolite profiles.

**Table 2:** Putatively Identified Compounds in Saffron Samples and Significance (*p* value <0.05) According to Supermarket, Group X or Y, or Adulteration

compound	RT	observed *m*/*z*	exact *m*/*z*	mass error (ppm)	ion	CCS value	empirical formula	mass fragments	group significance	proposed name	literature
1	3.34	503.1766	503.17701	–0.78	[M – H]^−^	157.96	C_22_H_32_O_13_	475.1816, 323.0963, 263.0749, 179.0709, 161.0434, 151.0742, 136.0507, 125.0233	supermarket	5-hydroxy-7,7-dimethyl-4,5,6,7-tetrahydro-3*H*-isobenzo-furanone-5-*O*-β-d-gentiobioside	Avula 2022^2^
2	3.37	489.1972	489.19775	–1.16	[M – H]^−^	158.21	C_22_H_34_O_12_	442.1642, 357.1533, 315.1424, 272.0861, 254.0752, 165.0902, 153.0899	supermarket	5-((3,4-dihydroxy-6-(((3,4,6-trihydroxy-5-(hydroxymethyl)oxan-2-yl)oxy)methyl)oxan-2-yl)oxy)-7,7-dimethyl-4,5,6,7-tetrahydro-2-benzofuran-1(3*H*)-one	
3	3.86	487.1817	487.1821	–0.97	[M – H]^−^	155.55	C_22_H_32_O_12_	425.1804, 385.1495, 343.1384, 325.1272, 181.0849, 180.0772, 153.0901, 125.0222	supermarket	5-((3,4-dihydroxy-6-(((3,4,6-trihydroxy-5-(hydroxymethyl)oxan-2-yl)oxy)methyl)oxan-2-yl)oxy)-7,7-dimethyl-4,7-dihydro-2-benzofuran-1(3*H*)-one	
4	6.55	329.232	329.23335	–4.03	[M – H]^−^	145.74	C_18_H_34_O_5_	267.0969, 251.1319, 233.2110, 229.1506, 211.1402, 193.0910, 183.1455, 171.1092, 165.1350, 139.1195, 127.1195, 116.9351	supermarket	(9*S*,10*S*,13*S*)-trihydroxy-(11*E*)-octadecenoic acid*	Cagliani 2015^18^
5	0.93	337.0764	337.07763	3.65	[M – H]^−^	132.51	C_12_H_18_O_11_	337.0764, 277.0547, 174.0148, 115.0014	X	2-*O*-α-d-glucopyranosyl-l-ascorbic acid*	
6	2.29	477.1613	477.1614	0.21	[M – H]^−^	165.76	C_20_H_30_O_13_	153.0538, 109.0637	Y	1-*O*-β-d-gentiobiosyl ester of 2-methyl-6-oxo-2,4-hepta-2,4-dienoic acid	Avula 2022^2^
7	3.07	1009.3793	1009.377	–2.28	[M – H]^−^	237.89	C_44_H_66_O_26_	1009.3793, 685.2717, 571.2387, 439.1436, 383.1438, 245.1148, 221.0642, 203.1418	Y	peroxidated form of α-crocin	Pham 2000^19^
8	3.59	813.3202	813.3186	–1.97	[M – H]^−^	325.95	C_38_H_54_O_19_	813.3202, 651.2661, 489.2126, 327.1586, 283.1687, 239.1787	X	1-*O*-[3,4,5-trihydroxy-6-(hydroxymethyl)oxan-2-yl] 16-*O*-[(6)-3,4,5-trihydroxy-6-[[3,4,5-trihydroxy-6-(hydroxymethyl)oxan-2-yl]oxymethyl]oxan-2-yl] (2,4,6,8,10,12,14)-2,6,11,15-tetramethylhexadeca-2,4,6,8,10,12,14-heptaenedioate	Avula 2022,^2^ Mena-Garcìa 2023^21^
9	3.89	473.1659	473.1664	1.06	[M – H]^−^	155.81	C_21_H_30_O_12_	473.1659, 305.0859, 283.1687, 167.0695, 125.0220	Y	4-allyl-2-hydroxyphenyl-6-*O*-β-d-glucopyranosyl-*O*-β-d-glucopyranoside	
10	4.49	975.3708	975.3714	0.62	[M – H]^−^	244.92	C_44_H_64_O_24_	651.2659	X	α-crocin isomer	Avula 2022^2^
11	4.58	685.2716	685.2713	–0.44	[M – H]^−^	197.05	C_32_H_46_O_16_	685.2716, 623.2692, 435.1183, 383.1441, 377.1588, 273.1465, 229.1575	Y	monohydroperoxide form of crocin 3	Pham 2000^19^
12	4.67	651.2661	651.2658	–0.46	[M – H]^−^	197.54	C_32_H_44_O_14_	651.2661, 489.2126, 327.1586, 283.1687, 239.1787	X	*trans*-crocetin gentiobiosylglucosyl ester (trans 3 Gg) degradation product	
13	4.69	813.3202	813.3186	–1.97	[M – H]^−^	230.97	C_38_H_54_O_19_	813.3202, 651.2661, 489.2126, 327.1586, 283.1687, 239.1787	X	*trans*-crocetin gentiobiosylglucosyl ester (trans 3 Gg)*	Avula 2022^2^
14	5.12	685.2716	685.2713	–0.44	[M – H]^−^	194.17	C_32_H_46_O_16_	685.2716, 665.2449, 493.3000, 473.2749, 447.0917, 383.1440, 361.1629, 341.1664, 311.2203, 297.1469, 273.1466, 253.0905, 229.1575	Y	monohydroperoxide form of crocin 3	Pham 2000^19^
15	5.22	1121.4217	1121.4293	6.78	[M – H]^−^	290.71	C_50_H_74_O_28_	1121.4217, 797.3035, 651.2661, 469.1344, 327.1586, 283.1687, 265.0697	X	(2,3,4,5,6)-6-((((2,3,4,5,6)-6-((((2,3,4,6)-3,4-dihydroxy-6-(hydroxymethyl)oxan-2-yl)oxy)methyl)-3,4,5-trihydroxyoxan-2-yl)oxy)methyl)-3,4,5-trihydroxyoxan-2-yl (2,3,4,5,6)-3,4,5-trihydroxy-6-((((2,3,4,5,6)-3,4,5-trihydroxy-6-(hydroxymethyl)oxan-2-yl)oxy)methyl)oxan-2-yl (2,4,6,8,10,12,14)-2,6,11,15-tetramethylhexadeca-2,4,6,8,10,12,14-heptaenedioate (crocin 6-OH)	
16	6.54	625.2502	625.2502	0.00	[M – H]^−^	205.99	C_30_H_42_O_14_	625.2502, 351.2139, 329.2318, 327.1575, 301.1428, 233.1136, 225.1265	X	Rubranoside D	
17	7.28	665.282	665.2814	–0.90	[M – H]^−^	223.26	C_33_H_46_O_14_	665.2820, 341.1746, 323.0967, 297.1844, 265.158, 153.0902	X	glycosylated crocin methyl ester	Si 2022^20^
18	7.28	1007.467	1007.47046	3.43	[M – H]^−^	288.68	C_47_H_76_O_23_	1007.467, 989.4558, 695.4657, 671.4651, 665.3524, 483.2714, 433.2349, 341.1753, 323.1469, 297.1845	X	hydrated and glycosylated form of 17	
19	3.59	1161.423	1161.4208	1.99	[M + Na]^+^	261.64	C_50_H_74_O_29_	1015.2632, 837.3156, 675.2623, 509.1477, 347.0954	X	crocin 6	Si 2022^20^
20	7.68	527.2248	527.2252	0.67	[M + Na]^+^	164.21	C_27_H_36_O_9_	365.1727, 185.0416	X	crocin 4	Si 2022^20^
21	7.49	367.1182	367.11871	–0.31	[M – H]^−^	203.69	C_21_H_20_O_6_	339.1996, 265.1475, 217.0501, 202.0262, 175.0398, 173.0606, 160.0162, 158.037, 149.0305, 134.0371, 132.0214	turmeric	cyclocurcumin	Jiang 2012^25^
22	3.17	609.1458	609.14611	–0.46	[M – H]^−^	225.88	C_27_H_30_O_16_	429.0827, 286.0414, 284.0321, 255.0294, 227.0344	saffron	quercetin-3-neohesperidinoside	Hegazi 2022,^4^ Mykhailenko 2022^26^
23	3.34	375.1657	375.16606	0.96	[M – H]^−^	192.62	C_17_H_28_O_9_	357.1497, 165.0914, 161.0448, 151.0764	saffron	(*Z*)-5-oxo-11-(β-d-glucopyranosyloxy)-8-undecenoic acid	Hegazi 2022^4^
24	9	379.1581	379.1564	4.57	[M – H]^−^	195.17	C_24_H_20_N_4_O	379.1581, 361.1473, 335.1677, 116.9282, 99.9253	paprika	Sudan IV	Monago-Maraña 2022^27^
25	4.14	975.3708	975.37148	0.70	[M – H]^−^	323.99	C_44_H_64_O_24_	651.2668, 533.1886, 473.2018, 323.0972	analytical standard	crocin 1	
26	6.01	651.2668	651.26583	–1.49	[M – H]^−^	197.54	C_32_H_44_O_14_	651.2668, 327.1586	analytical standard	crocin 3	

To better understand the quality of the saffron samples, S-plots between samples that originate from the same geographical area were calculated to identify the markers responsible for the difference (Figures S2–S4). In fact, in many cases, samples from the same geographical area were in different regions of the PCA. Surprisingly, the metabolites responsible for the separation along PC1 were always the same. Thus, we checked the OPLS-DA between the group at the right (A, E, F, G, L) and the one at the left (all others) of the PCA to confirm this hypothesis (Figure S5), and the significant *m*/*z* are reported in Table 2.

**Figure 3:**
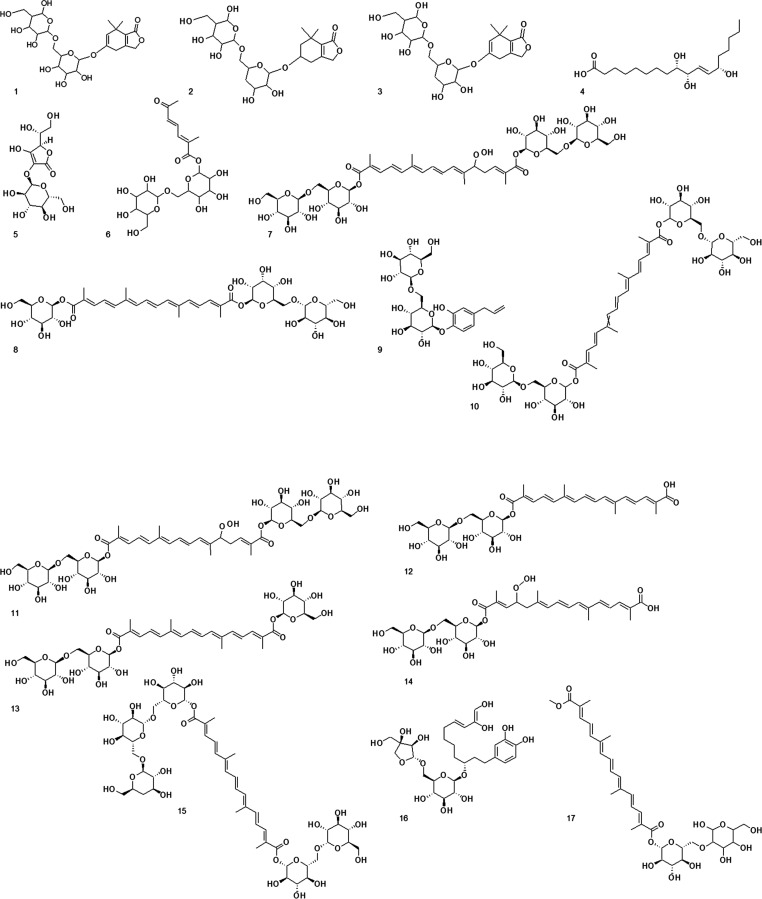

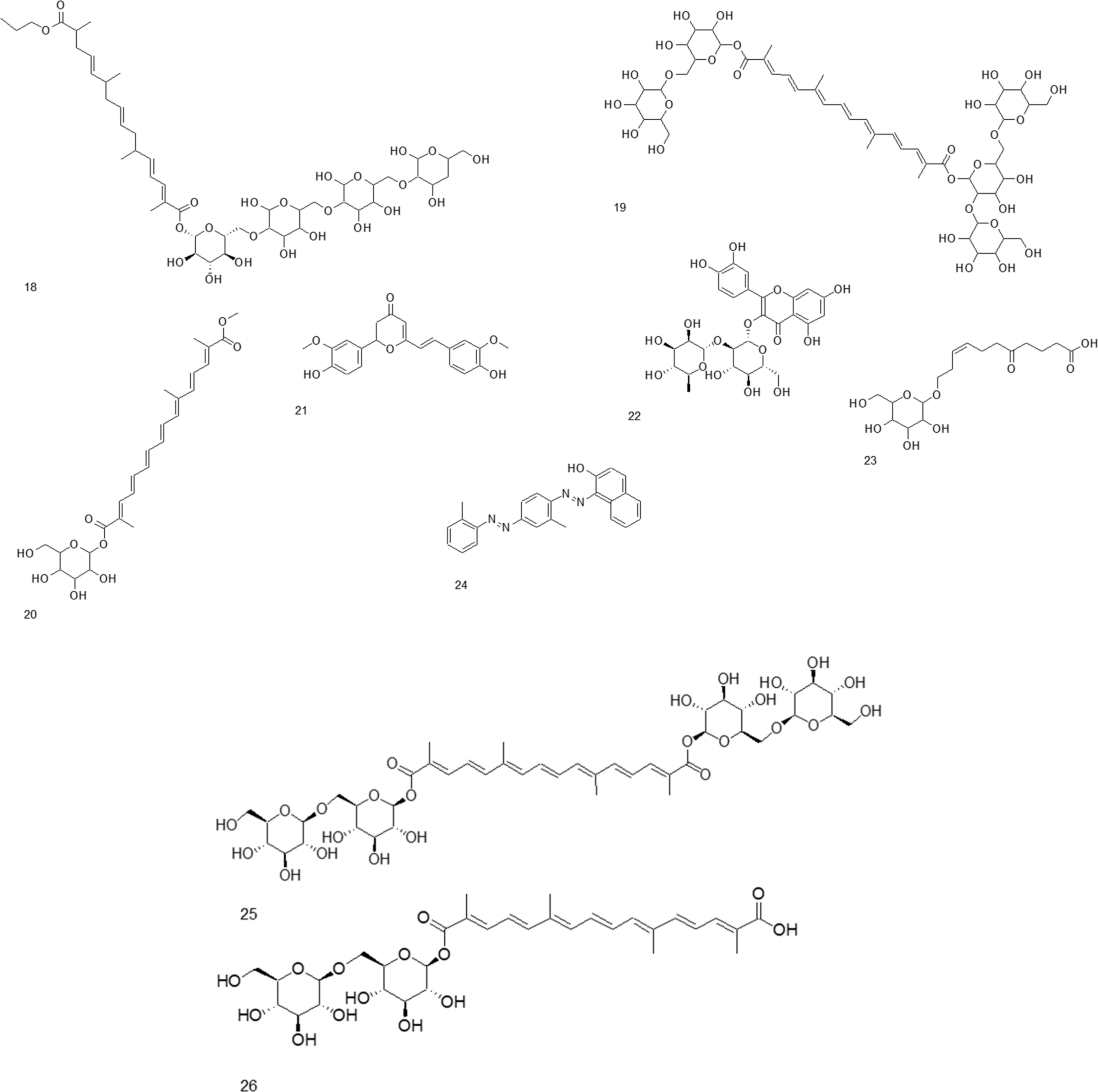
Chemical structures for compounds **1**–**26**. **1**–**4**, **6**–**12**, and **14**–**23** are proposed based on MS data or/and literature data, and the stated stereochemistries are tentatively deduced in accordance to the known compounds in the literature.

The proposed structures for the compounds are reported in Figure 3.

We used UPLC-ESI-TOF MS techniques to investigate the metabolome of saffron and identified several compounds that are crucial for assessing its quality and authenticity.

In detail, compound **5** was unequivocally identified as 2-*O*-α-d-glucopyranosyl-l-ascorbic acid. This identification was achieved by comparison of the fragmentation pattern and RT with the corresponding analytical standard.

Compound **6** was tentatively identified as 1-*O*-β-d-gentiobiosyl ester of 2-methyl-6-oxo-2,4-hepta-2,4-dienoic acid. The detailed mass spectrum analysis revealed characteristic losses such as the 3-penten-2-one moiety (C_5_H_8_O, *m*/*z* 393.0594). Also, its base peak at *m*/*z* 153.0538 corresponded to the neutral loss of the gentiobiose moiety (C_12_H_22_O_10_). Finally, the fragment at *m*/*z* 109.0637 corresponded to the neutral loss of carbonyl gentiobiose (C_13_H_20_O_12_). This compound was already reported in the literature for saffron, which made our hypothesis more reliable.^2^

The precursor ion and fragmentation pattern of compound **7** highlighted its similarity to α-crocin (see compound **11**), with the addition of a hydroperoxide moiety, which is a modification likely derived from oxidative processes similar to those observed in lipid peroxidation. A similar compound for monohydroperoxide-alpha-crocin was reported by Pham (2000) et al.,^19^ but several isomeric structures could be proposed according to the position of the oxidation site. Specifically, *m*/*z* 685.2717 corresponded to the neutral loss of the gentiobiosyl moiety (C_12_H_21_O_10_), *m*/*z* 571.2387 corresponded to the neutral loss of C_17_H_26_O_13_, *m*/*z* 439.1436 corresponded to the loss of C_23_H_39_O_16_, *m*/*z* 323.0963 corresponded to the loss of C_28_H_44_O_17_, *m*/*z* 221.0642 corresponded to the loss of C_36_H_51_O_17_, and *m*/*z* 115.0378 corresponded to the loss of C_39_H_56_O_21_.

Based on the accurate mass data and fragmentation pattern, compounds **13** and **8** were, respectively, identified as crocin 2 isomers. In detail, compound **13** was confirmed as *trans*-crocetin gentiobiosylglucosyl ester (also known as crocin 2 or trans 3 Gg) after cochromatography with the analytical standard. Compound **8** instead was speculated to be an isomer of *trans*-crocetin ester gentiobiosylglucoside due to the missing diagnostic fragment at *m*/*z* 485.15 typical of crocetin esters containing the triglucosyl moieties. Moreover, the missing peak at 325 nm in the UV–vis data excluded the possibility of a *cis*-crocetin ester for **8**.^2,20^ Therefore, compound **8** was proposed to be 1-*O*-[3,4,5-trihydroxy-6-(hydroxymethyl)oxan-2-yl]16-*O*-[(6)-3,4,5-trihydroxy-6-[[3,4,5-trihydroxy-6-(hydroxymethyl)oxan-2-yl]oxymethyl]oxan-2-yl] (2,4,6,8,10,12,14)-2,6,11,15-tetramethylhexadeca-2,4,6,8,10,12,14-heptaenedioate. Compound **9** was tentatively identified as 4-allyl-2-hydroxyphenyl-6-*O*-β-d-glucopyranosyl-*O*-β-d-glucopyranoside based on mass data, even if there is no evidence in the literature of the presence in saffron of this compound.

**Figure 4:**
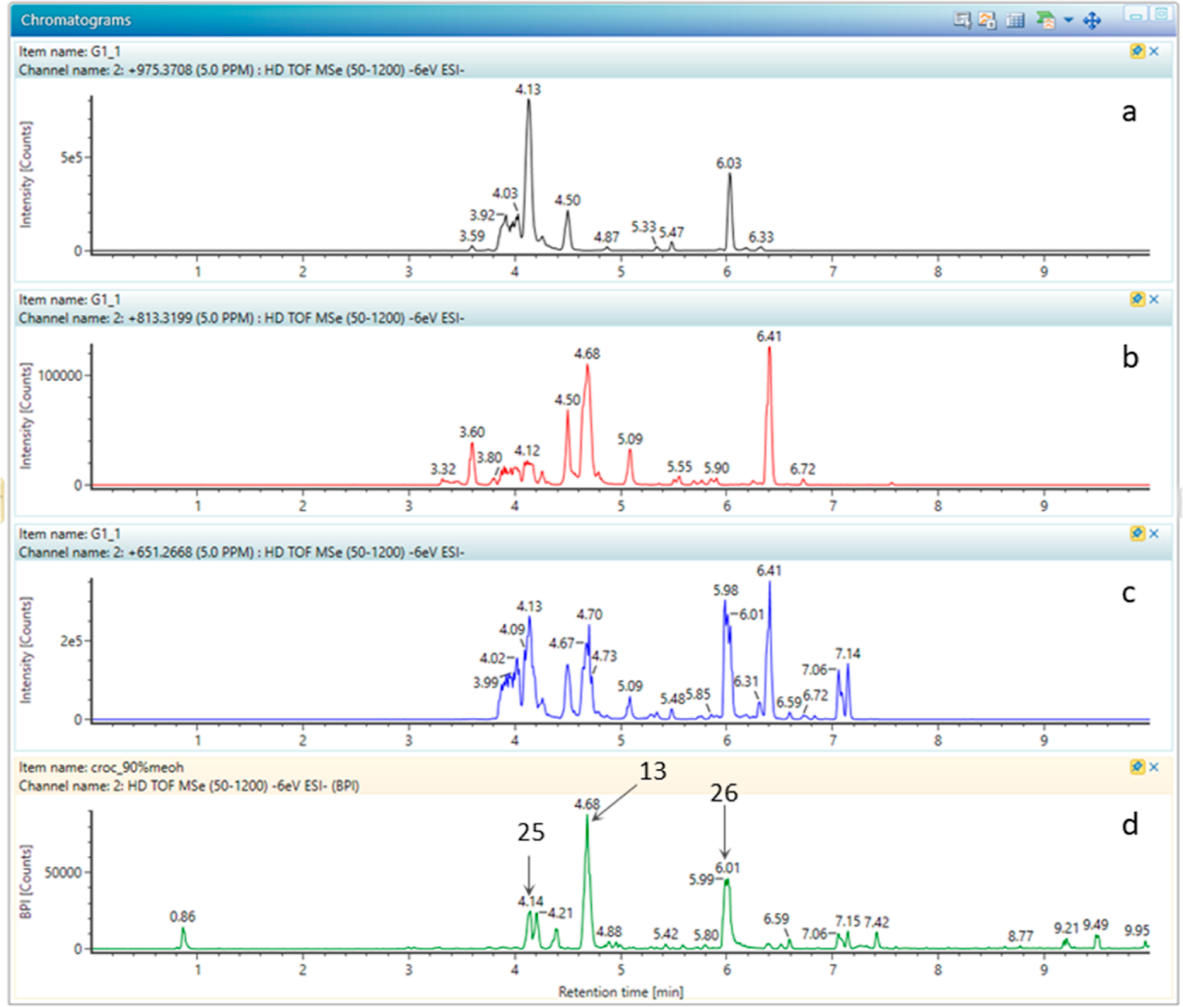
Extracted ion chromatogram of crocin 1 (a), crocin 2 (b), and crocin 3 (c) isomers in saffron sample G, and reference compounds crocin 1 (**25**), 2 (**13**), and 3 (**26**) (d).

Compound **10** had the same sum formula and fragmentation pattern as crocetin digentiobiosyl ester or alpha-crocin (*trans*-4-GG), which eluted at 4.14 min (confirmation with cochromatography with the corresponding analytical standard). Since from the UV–vis no peaks at 325 nm were detected, it was speculated to be a *trans*-4-GG isomer. As expected, we found many isomeric forms for crocins that hampered the correct annotation of the compounds. For this reason, injecting analytical standards was crucial to determine the correct assignment of the main crocins. Indeed, Figure 4 shows the extracted ion chromatograms of alpha-crocin (a), crocin 2 (b), and crocin 3 (c) isomers in a saffron sample and the reference compounds (d). The fragmentation pattern and molecular structure for crocins 1–3 (**25**), (**13**), and (**26**) are reported in Table 2 and Figure 3.

**Figure 5:**
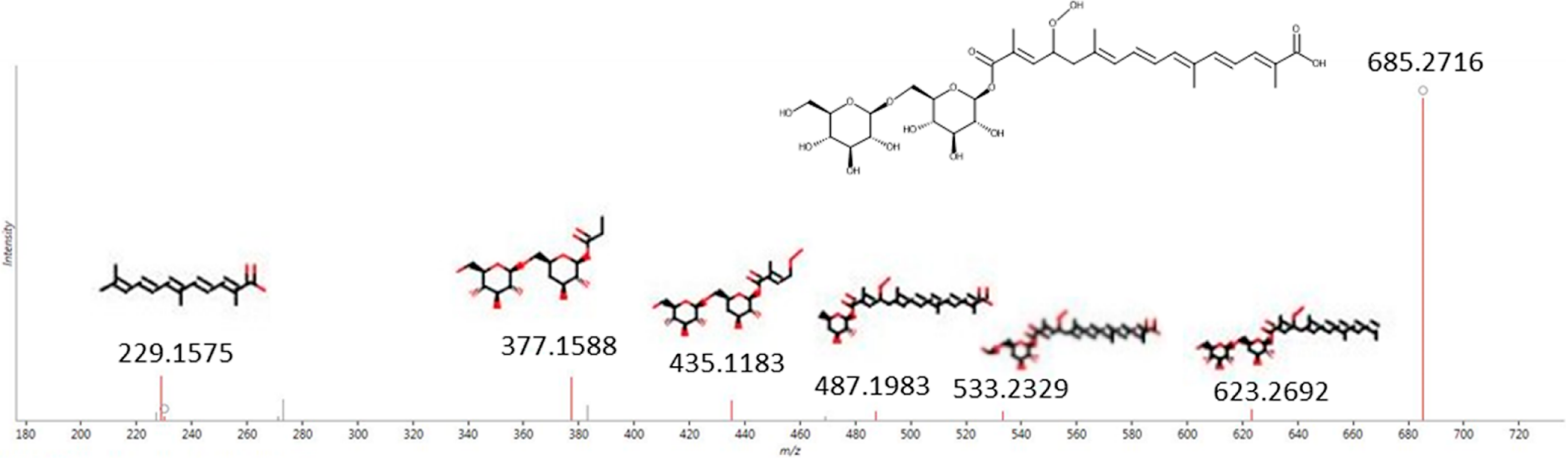
High-collision energy (MS^e^) fragmentation spectrum of compound **14**.

Compounds **11** and **14** were tentatively identified as monohydroperoxide forms of crocin 3. Several structures could be proposed according to the position of the oxidation site. However, the diagnostic fragment at *m*/*z* 229.1575 allowed the assignment of compound **11** specifically to the structure reported in Figure 5. Compound **14** instead indicated in the spectrum some fragments typical for compound **17**; therefore, a specific assignment could not be possible.

Compound **12** was clearly a degradation product of compound **13** that lost a sugar moiety and was already described in saffron samples.^21^

Metabolite **15** had the same molecular formula and fragmentation pattern as that of crocin 6, but without a hydroxyl group. Such compound was not previously documented in the scientific literature. This finding led to a search for other crocetin esters that were missing a hydroxyl group in the mass spectrometry data. Surprisingly, these compounds were identified at retention times (RT) distinct from those of known compounds, as detailed in Figure S6. The proposed IUPAC name was (2,3,4,5,6)-6-((((2,3,4,5,6)-6-((((2,3,4,6)-3,4-dihydroxy-6-(hydroxymethyl)oxan-2-yl)oxy)methyl)-3,4,5-trihydroxyoxan-2-yl)oxy)methyl)-3,4,5-trihydroxyoxan-2-yl (2,3,4,5,6)-3,4,5-trihydroxy-6-((((2,3,4,5,6)-3,4,5-trihydroxy-6-(hydroxymethyl)oxan-2-yl)oxy)methyl)oxan-2-yl (2,4,6,8,10,12,14)-2,6,11,15-tetramethylhexadeca-2,4,6,8,10,12,14-heptaenedioate.

Rubranoside D could be proposed for compound **16**, a component described in *Alnus rubra*.^22,23^ Since the matching with the in-silico fragments gave a low score and this compound has never been described in saffron before, the identification needs to be further proven via a reference compound or isolation.

Fragmentation spectra and RT of compound **17** corresponded to a glycosylated crocin methyl ester, a new type of crocin characterized recently.^20^

Finally, compound **18** was attributed to a hydrated and glycosylated form of **17** since the fragmentation pattern was very similar. A clear structure could not be proposed, but the higher collision cross section (CCS) value for **18** indicated that the molecular formula is higher than that of **17**, even if they coeluted. Isolation and nuclear magnetic resonance (NMR) spectroscopy would be needed in future studies to describe the molecular structure.

The score plot obtained from the UPLC-ESI-TOF MS data in the positive ESI mode confirmed the separation between the samples obtained in the negative ionization mode. Moreover, it further highlights the impact of crocins in the separation between groups X and Y. Indeed, two crocins, different from the previous ones mentioned above, were putatively identified through the mass data. In detail, *m*/*z* 1161.4230 at a RT of 3.59 min and *m*/*z* 505.2443 at RT of 7.68 min. The first ion corresponded to the precursor ion [M + Na]^+^ and was assigned to a crocin 6 isomer (**19**) presenting the diagnostic fragments at *m*/*z* 1015.2632, 837.3156, 675.2623, 509.1477, and 347.0954. The second ion corresponded to the precursor ion [M + H]^+^ and was assigned to crocin 4 (**20**) thanks to the diagnostic fragments at *m*/*z* 365.1727 and 185.0416.

Consequently, crocins represent a promising tool to screen and establish saffron quality in the Italian market. All of the spectra of compounds **1–26** are reported in the SI in Figures S7–S32.

### 3.3 Markers to Identify Turmeric and Paprika Adulterations of Saffron

In this section, we explore the challenges of distinguishing authentic saffron samples from those adulterated with turmeric and paprika. As no difference in geographical origin was detected, it could be difficult to distinguish an authentic saffron sample from an adulterated one.

Using advanced UPLC-ESI-TOF MS techniques in the positive ESI mode, we analyzed a saffron quality control blend adulterated with different percentages (10, 25, and 50%) of turmeric and paprika.^24^ Our analysis, visualized by PCA (e.g., Figure S33), showed clear separations even at the lowest level of adulteration (10%), highlighting the sensitivity of the technique.

Significant markers for turmeric and saffron were identified from the S-plots (Figure S34). In detail, for turmeric, the significant signal of *m*/*z* 367.1182 increased along with the increasing of adulterant percentage, as depicted in the trend plot (Figure S35). According to mass data, *m*/*z* 367.1182 eluted at 7.49 min, and the sum formula C_21_H_20_O_6_ was calculated. The diagnostic fragments at *m*/*z* 339.1996, 265.1475, 217.0501, 202.0262, 175.0398, 173.0606, 160.0162, 158.037, 149.0305, 134.0371, and 132.0214 allowed the assignment to cyclocurcumin (**21**). This is one of the most important metabolites of *C. longa*.^25^

Moreover, it was possible to individualize markers of saffron authenticity, in particular, *m*/*z* 609.1458. The metabolite eluted at 3.17 min and the elemental composition of C_27_H_30_O_16_ was calculated. By means of the diagnostic fragments of *m*/*z* 429.0827, 286.0414, 284.0321, 255.0294, and 227.0344, it was possible to attribute the *m*/*z* to quercetin-3-neohesperidinoside (**22**). The structure could be distinguished from a glycosylated kaempferol via the fragment at *m*/*z* 227 and was already found in saffron samples.^4,26^

Another significant *m*/*z* for saffron indicated by the S-plots was 375.1657. The metabolite eluted at 3.34 min, and the formula of C_17_H_28_O_9_ was calculated. The diagnostic fragments at *m*/*z* 357.1497, 165.0914, 161.0448, and 151.0764 allowed a tentative identification as (*Z*)-5-oxo-11-(β-d-glucopyranosyloxy)-8-undecenoic acid (**23**), previously reported in saffron.^4^

The results obtained for paprika adulteration were similar to those for turmeric. Indeed, also in this case, the score plots of all the adulterated samples indicated along PC2 a separation already with the addition of 10% of adulterant (Figure S36). Again, the most significant markers for saffron according to the S-plot were *m*/*z* 609.1458, already attributed to quercetin-3-neohesperidinoside, and *m*/*z* 337.0763, 2-*O*-α-d-glucopyranosyl-l-ascorbic acid (**1**) (Figure S37). Concerning paprika, the most significant metabolite was *m*/*z* 379.1581, which eluted at 9.2 min (Figure S38, trend plot). The diagnostic fragments were *m*/*z* 379.1581, 361.1473, 335.1677, 116.9282, and 99.9253, and the proposed identification was Sudan IV (**24**) known as paprika adulterant.^27^

**Figure 6:**
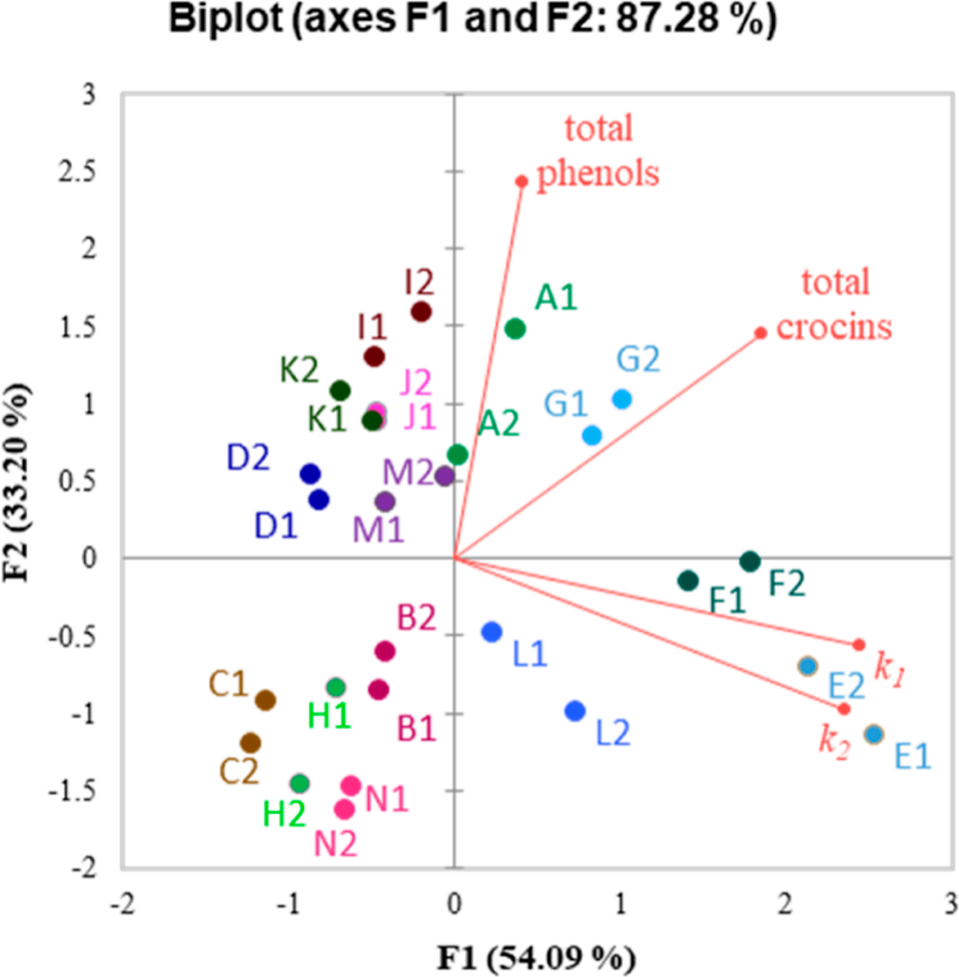
Score plot (comp 1 vs comp 2) of total phenol content (TPC), total crocin content, and kinetic parameters for the antioxidant activity.

To better describe the chemical quality of the saffron extracts, the total crocin content, the total phenolic content, and the antioxidant activity with a novel kinetic-based DPPH^•^ method were determined. The total crocin content correlated with the results obtained from the S-Plot. Samples A, E, F, and G had higher total crocins than the others, as reported in Table S2. As highlighted in the score plot in Figure 6, analysis of each saffron sample showed rather good clustering of each replicate in close proximity, confirming the reproducibility of the techniques.

Inspection of the PCA output illustrated that the largest differences were observed between samples according to all of the parameters investigated along PC1. Information regarding the eigenvectors and eigenvalues is reported in Figure S38. The highest amount of phenols was reported in sample I, while the lowest in sample N. Samples E and F, classified within the high-crocin category, also showed the greatest antioxidant activity in terms of *k*_1_ and *k*_2_, followed by samples M, N, and B (Table 1).

Overall, the results illustrated that the samples discovered to be higher in crocins are differentiated for antioxidant parameters. Thus, the higher quality of these saffron samples was demonstrated also by considering the antioxidant properties of their composition.

In conclusion, the untargeted metabolomics approach using UPLC-ESI-TOF MS was able to provide a fast and first insight into the composition and differences among the saffron samples. Potential markers for saffron purchased at a supermarket were found, such as (9*S*,10*S*,13*S*)-trihydroxy-(11*E*)-octadecenoic acid (group Y) and also markers allowing a further discrimination on saffron quality, such as 2-*O*-α-d-glucopyranosyl-l-ascorbic acid (**5**) and crocins (**8**, **10**, **12**, **13**, **15**, and **17**–**20**) for the higher quality saffron sample group (group X) and, on the other hand, oxidation products of crocins for group Y. Although a discrimination regarding geographical origins was not possible, adulteration with turmeric and paprika at a percentage of 10% was successfully detected, by putatively identifying specific markers, i.e., respectively, cyclocurcumin and Sudan IV. Moreover, several approaches to study the in vitro antioxidant activity of the extracts were performed, validating the UPLC-ESI-TOF MS approach and thus confirming that the same samples identified as richer in crocins also showed higher antioxidant properties in terms of phenolic content, total crocin content, and DPPH^•^ kinetics.
