# Supplementary material for: UPLC-ESI-TOF MS Profiling Discriminates Biomarkers in Authentic and Adulterated Italian Samples of Saffron (Crocus sativus L.)
Source: ACS Food Sci Technol. 2024 Jul 4;4(7):1783–94. doi: 10.1021/acsfoodscitech.4c00340 (PMC11265267; doi:10.1021/acsfoodscitech.4c00340)
Supplement: Supplementary file 1 — fs4c00340_si_001.pdf [file fs4c00340_si_001.pdf]

Supporting Information

UPLC-ESI-TOF MS profiling discriminates biomarkers in authentic and adulterated Italian samples of saffron (*Crocus sativus L.*)

Lucrezia Angeli[1], Ksenia Morozova[1], Corinna Dawid,[2,3] Matteo Scampicchio[1], and Timo D. Stark[3*]

[1]*Faculty for Agricultural, Environmental, and Food Sciences, Free University of Bozen-Bolzano, 39100, Italy*

[2]*Professorship for Functional Phytometabolomics, TUM School of Life Sciences, Technical University of Munich, Lise-Meitner-Str. 34, 85354 Freising, Germany*

[3]*Food Chemistry and Molecular Sensory Science, Technical University of Munich, Lise-Meitner-Str. 34, 85354 Freising, Germany*

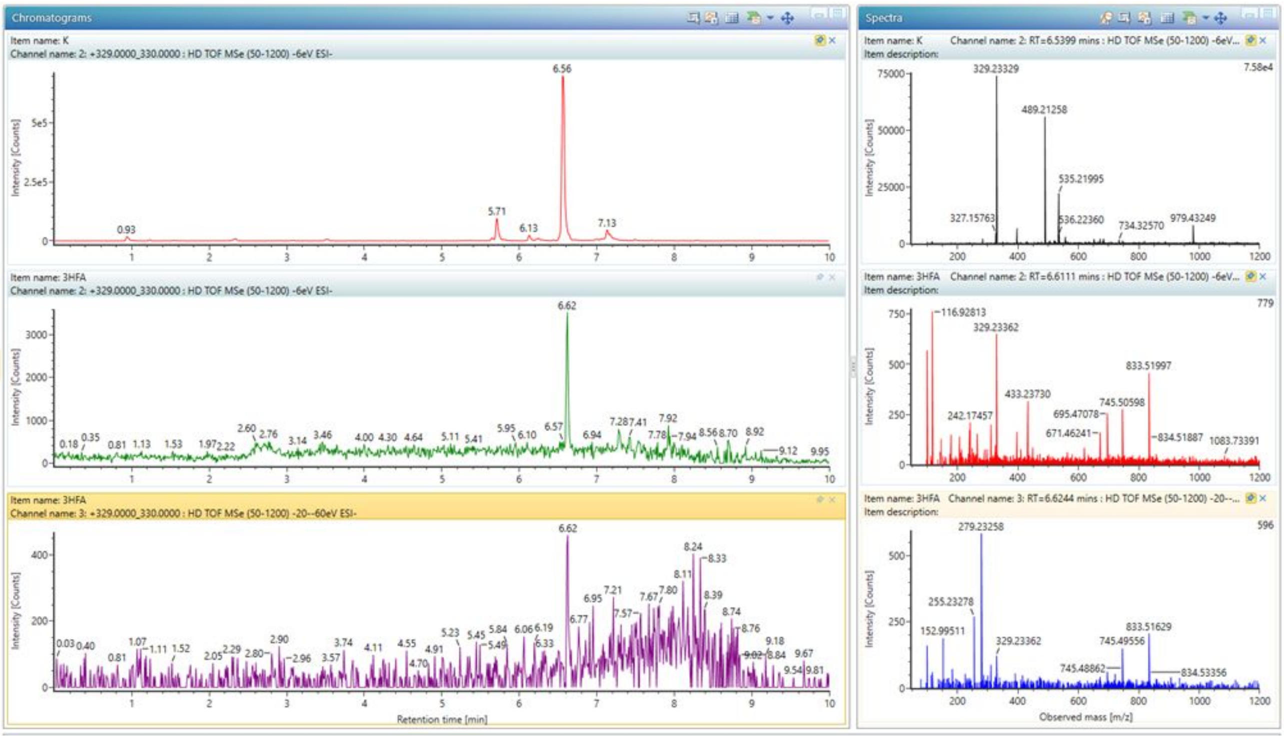

Fig. S1. Confirmation of 9(S)-,-10-(S)-,-13-(S)-Tri-hydr-oxy-11-(E)-octa-decen-oic acid with the analytical standard injection.

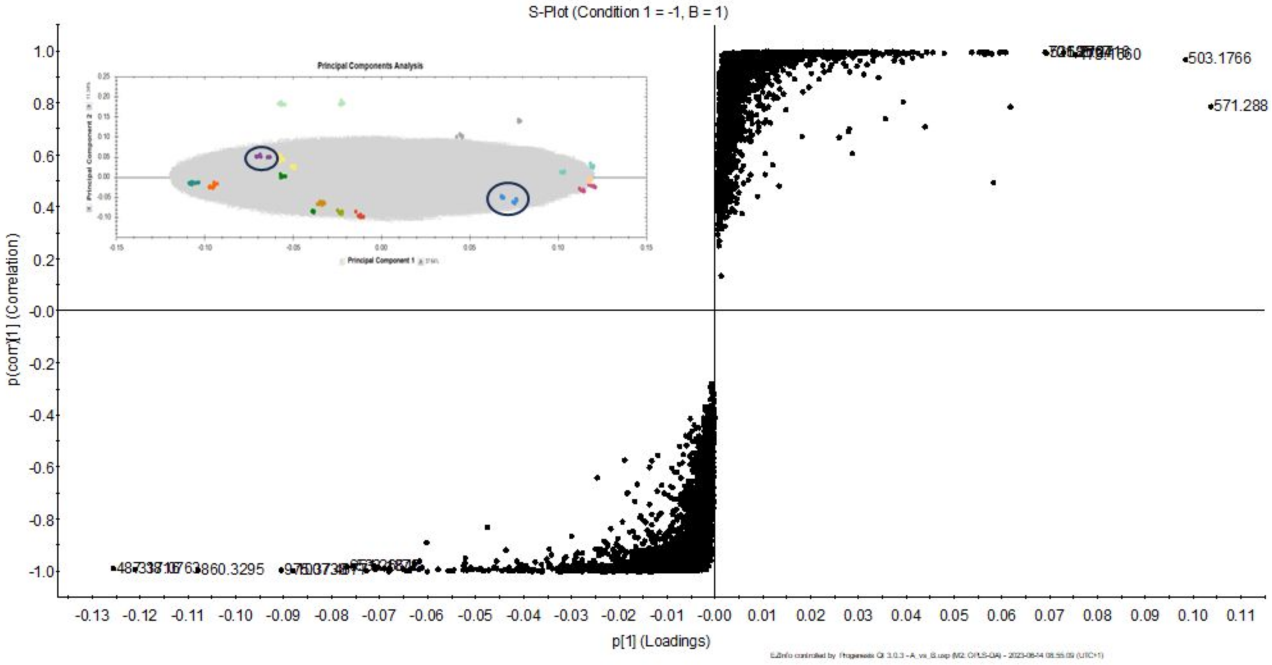

Fig. s2. S-Plot between samples A and B, both from Trentino-Alto Adige, a region in the north of Italy.

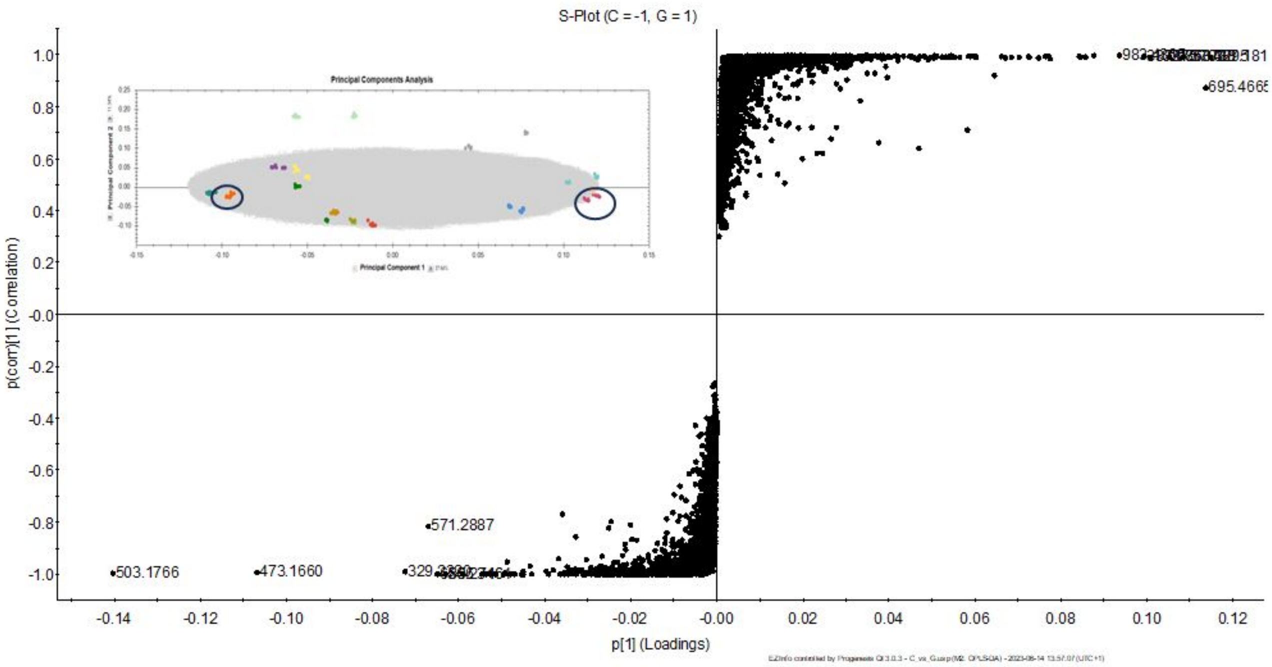

Fig. s3. S-Plot between samples C and G, both from an area in the north-west of Italy.

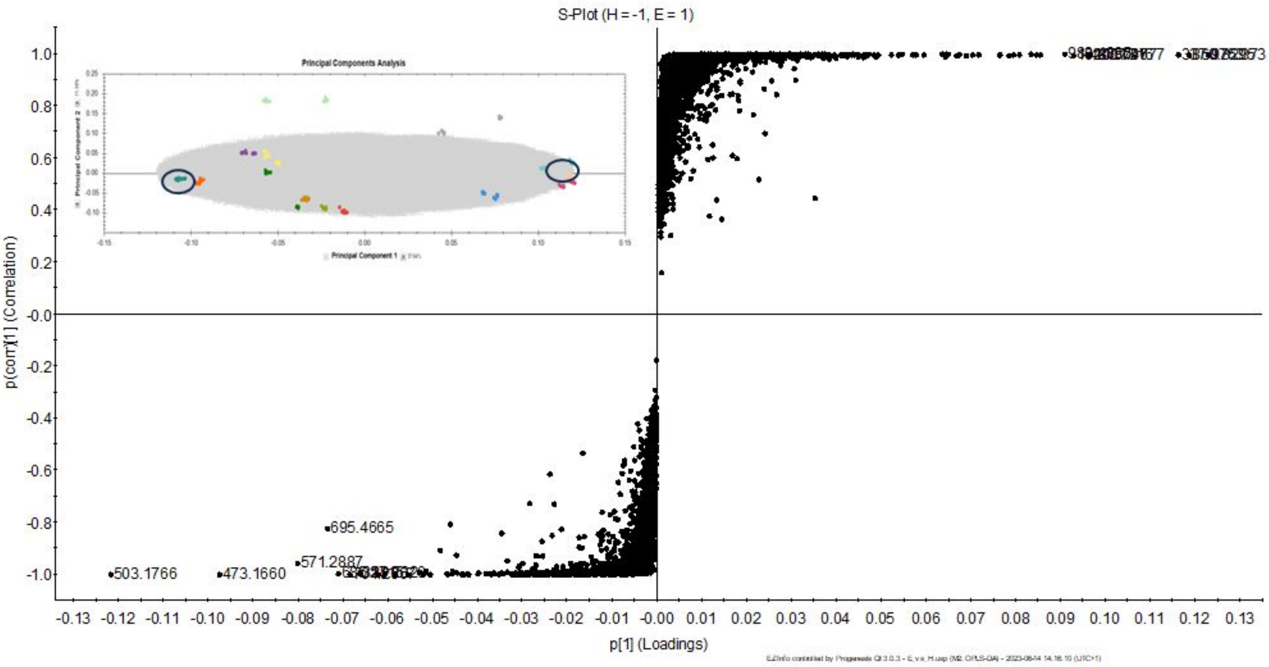

Fig. s4. S-Plot between samples H and E, both from an area in the centre of Italy.

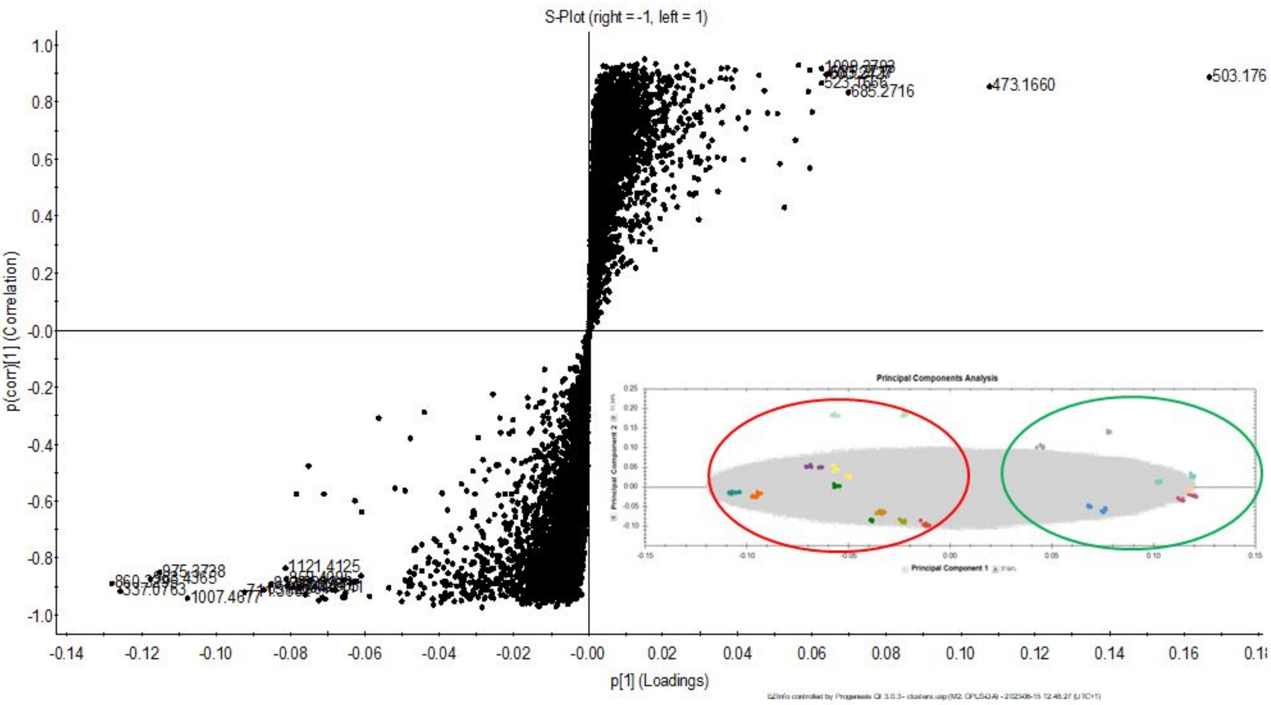

Fig. s5. S-Plot and score plot between the two bigger clusters visible.

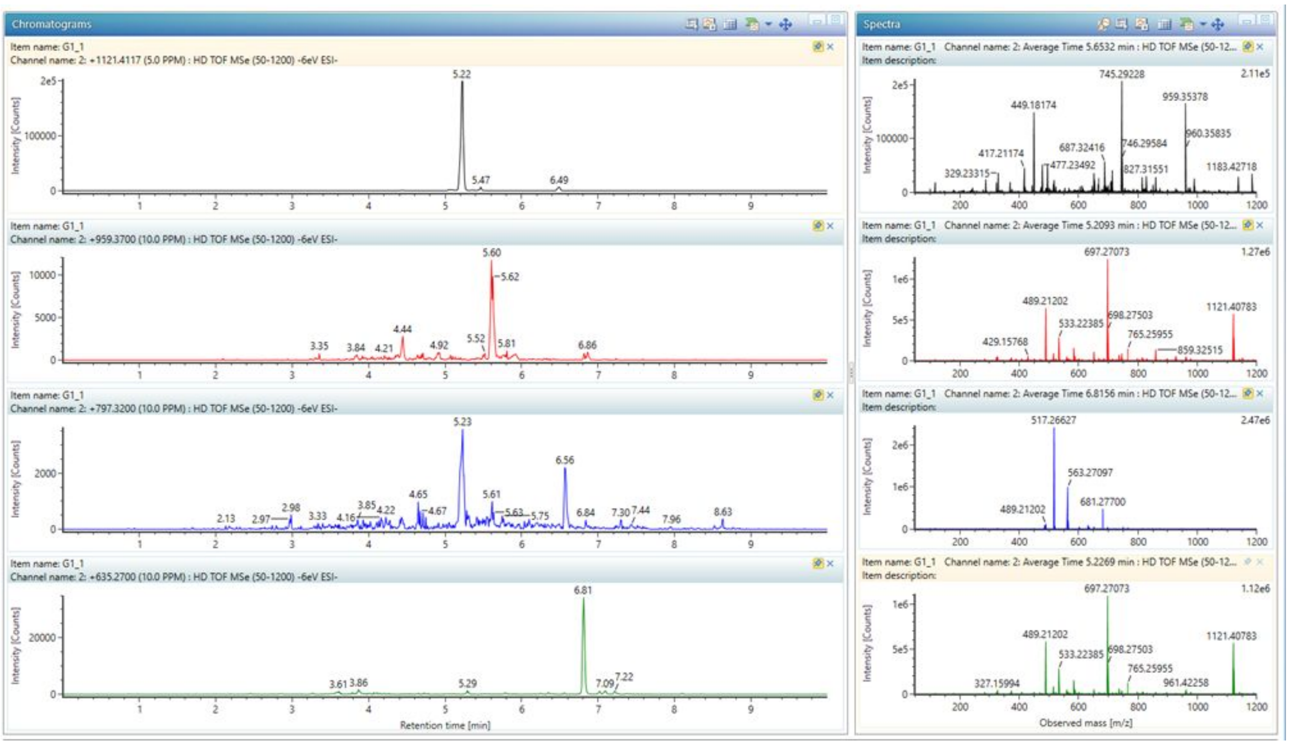

Fig. s6. BPI and fragmentation spectra of de-hydroxylated crocins.

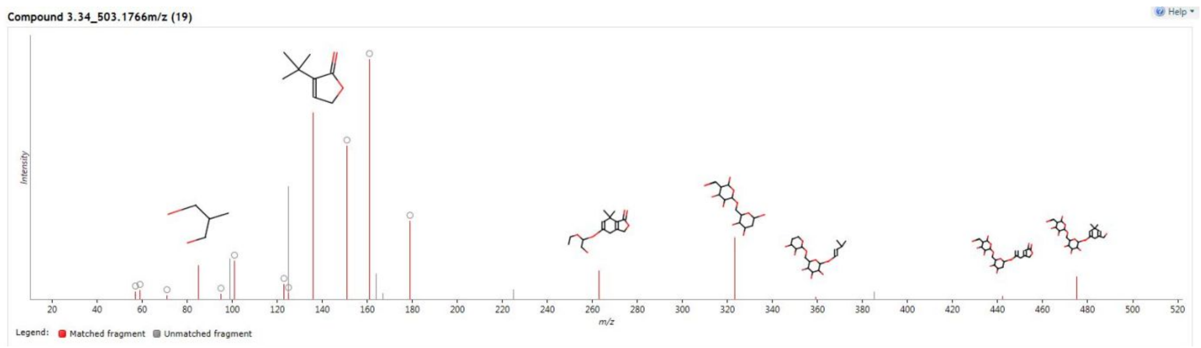

Fig. s7. Fragmentation spectrum of compound 1.

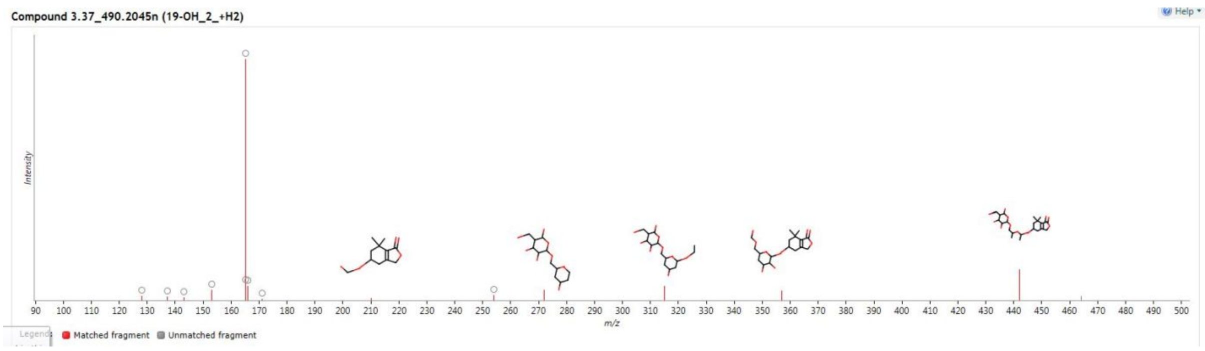

Fig. s8. Fragmentation spectrum of compound 2.

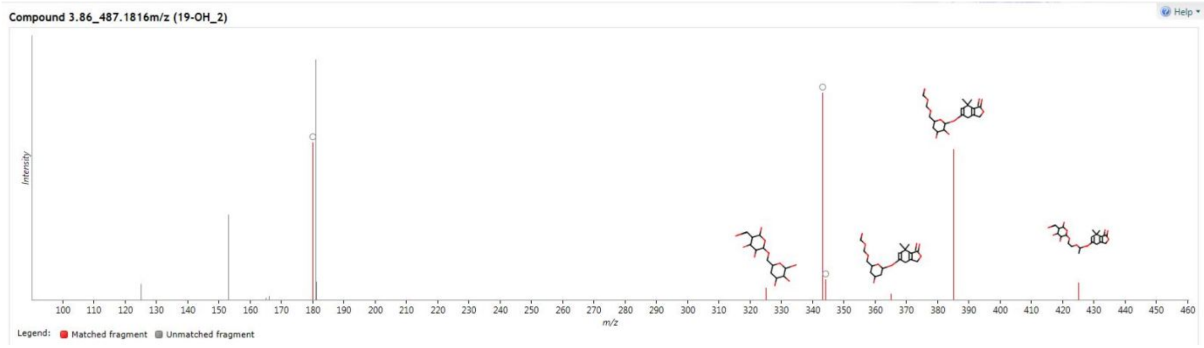

Fig. s9. Fragmentation spectrum of compound 3.

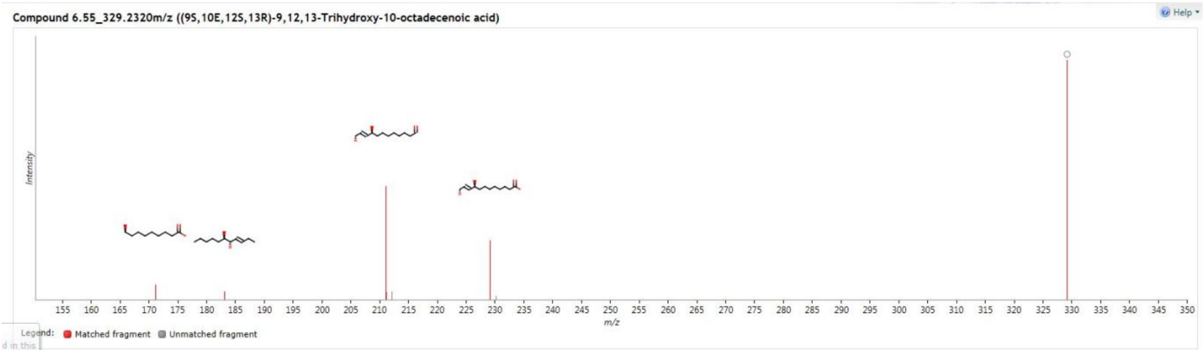

Fig. s10. Fragmentation spectrum of compound 4.

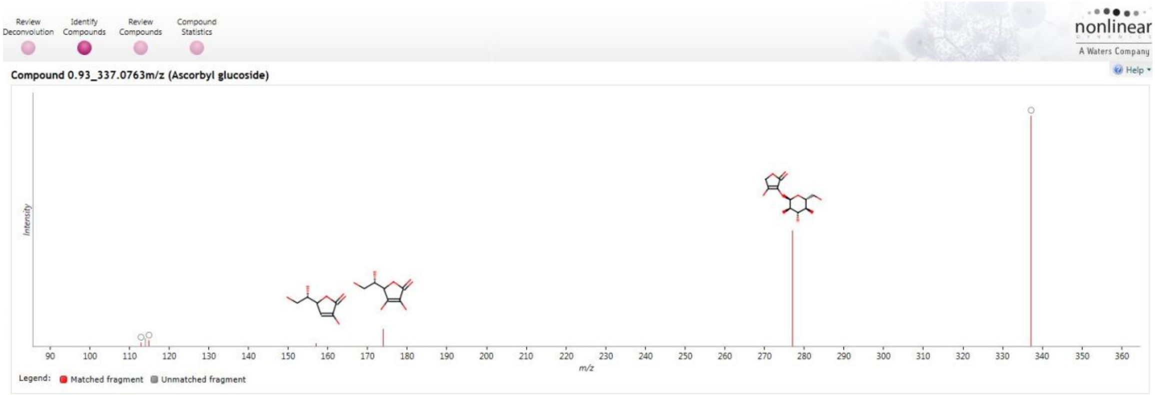

Fig. s11. Fragmentation spectrum of compound 5.

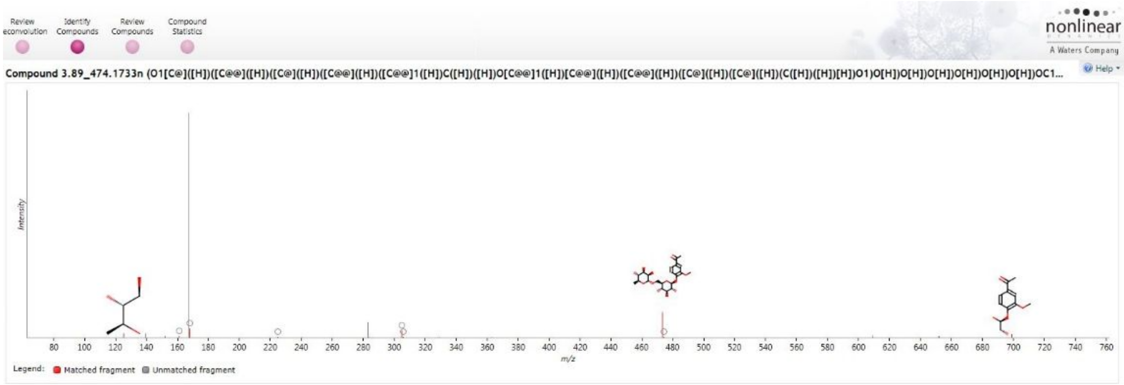

Fig. s12. Fragmentation spectrum of compound 6.

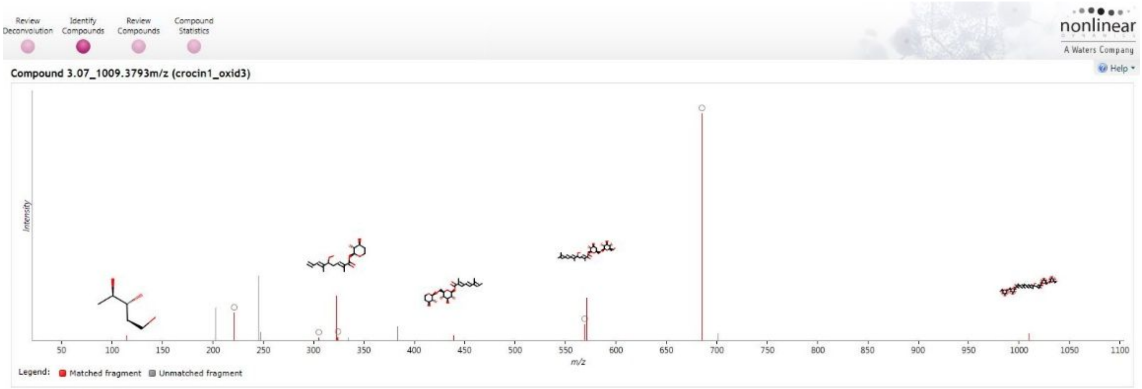

Fig. s13. Fragmentation spectrum of compound 7.

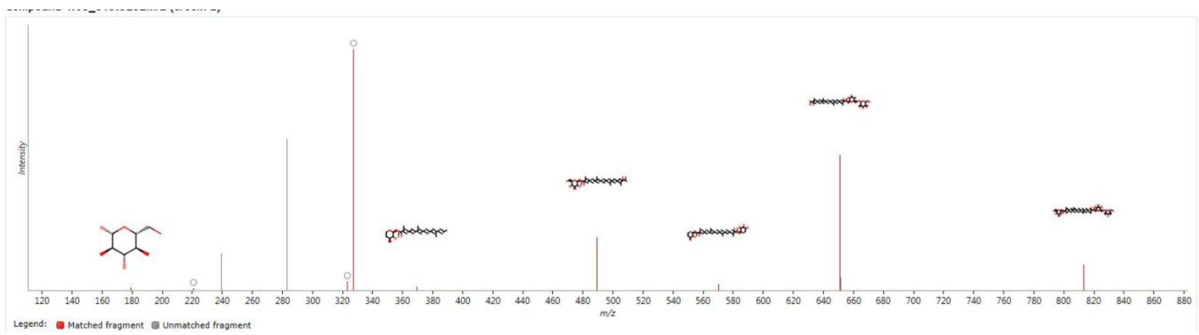

Fig. s14. Fragmentation spectrum of compound 8.

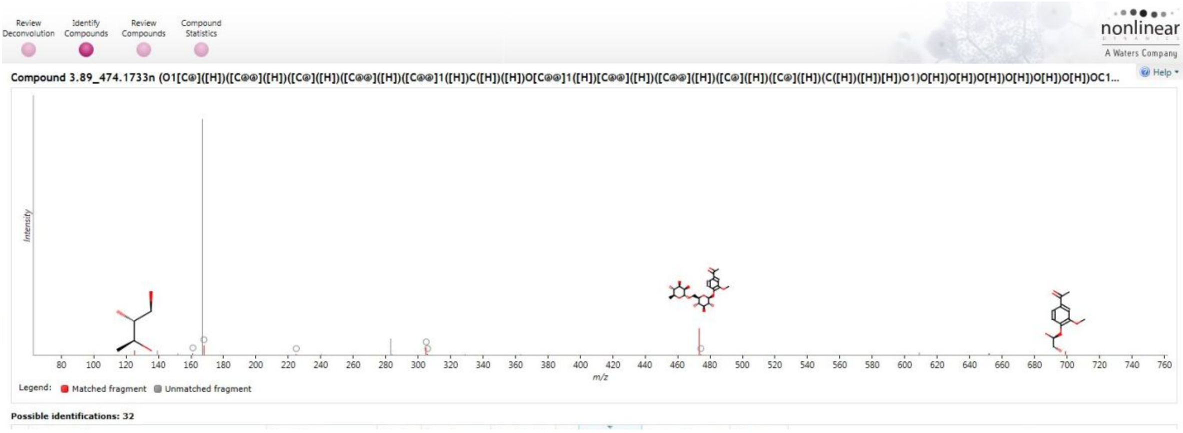

Fig. s15. Fragmentation spectrum of compound 9.

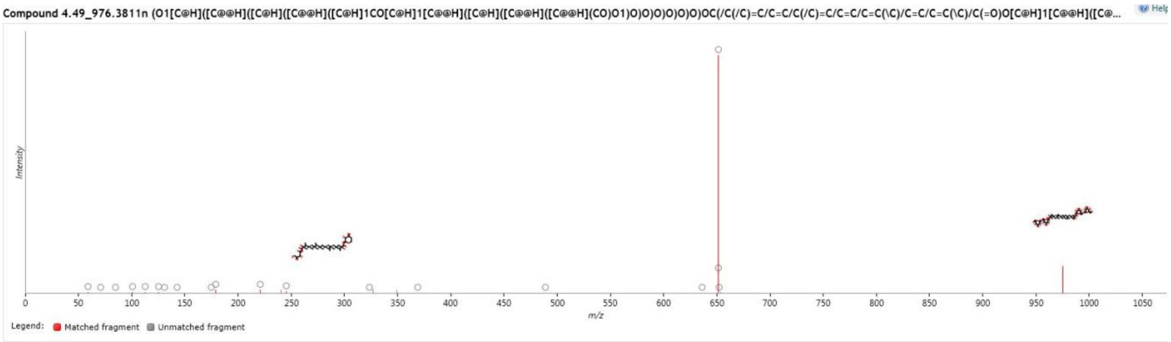

Fig. s16. Fragmentation spectrum of compound 10.

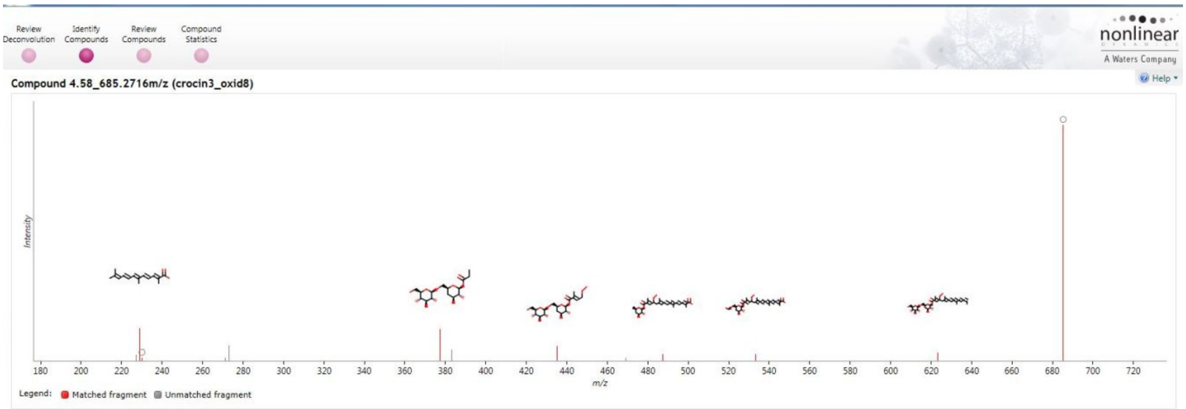

Fig. s17. Fragmentation spectrum of compound 11.

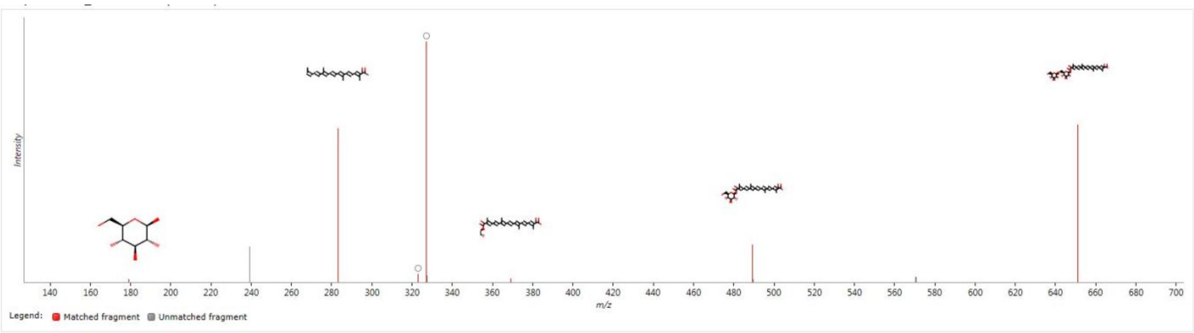

Fig. s18. Fragmentation spectrum of compound 12.

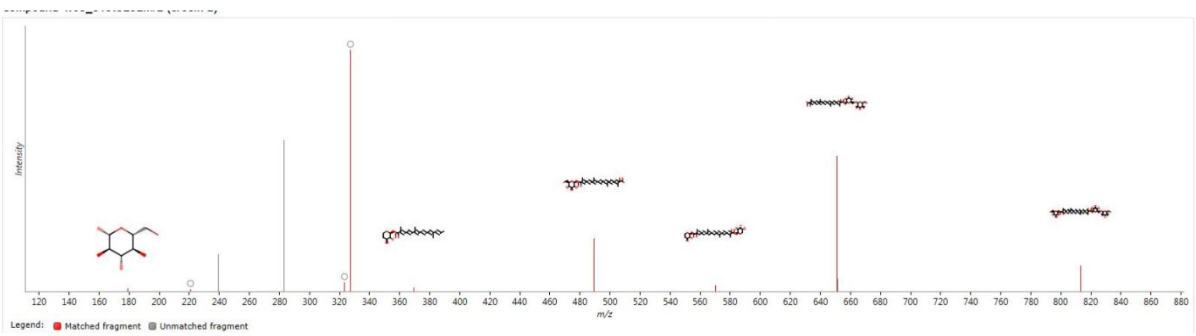

Fig. s19. Fragmentation spectrum of compound 13

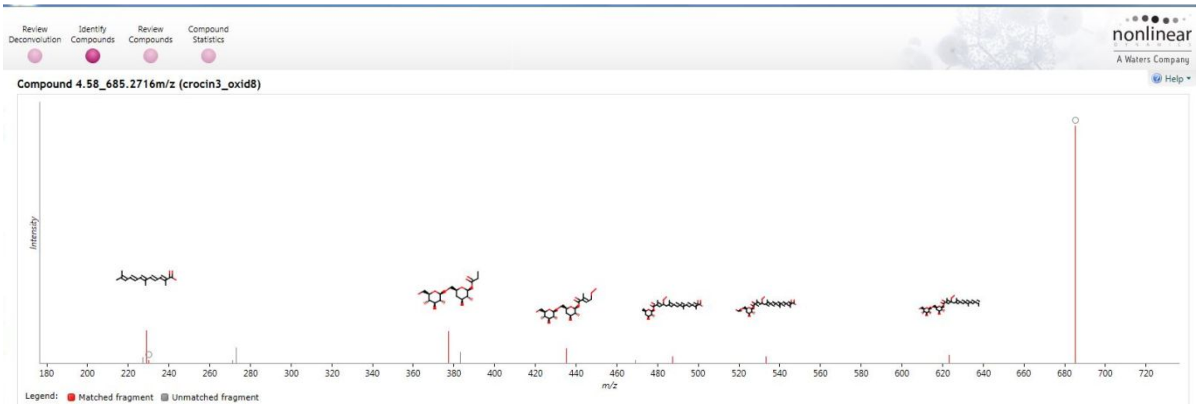

Fig. s20. Fragmentation spectrum of compound 14.

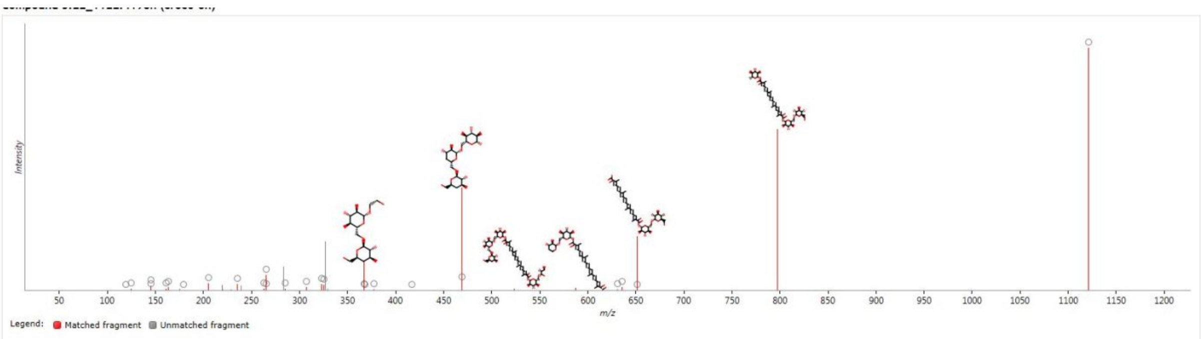

Fig. s21. Fragmentation spectrum of compound 15.

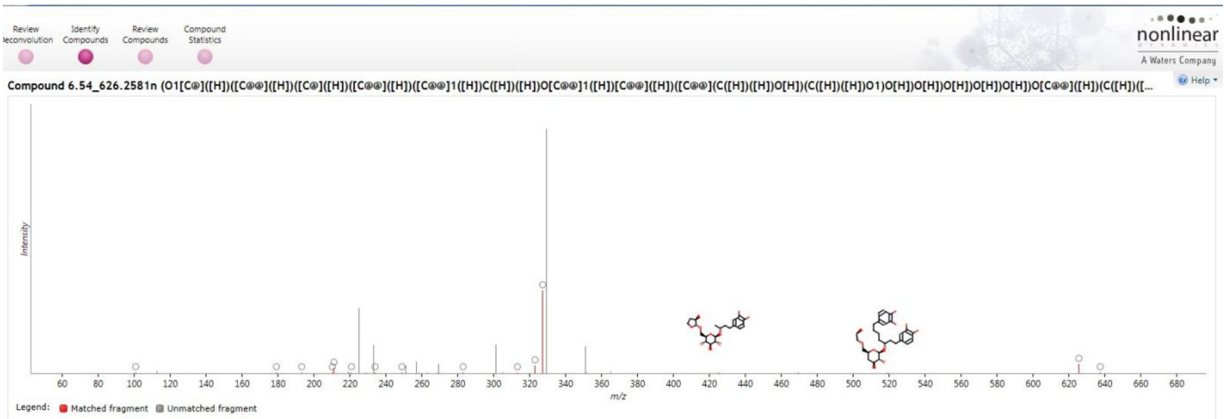

Fig. s22. Fragmentation spectrum of compound 16.

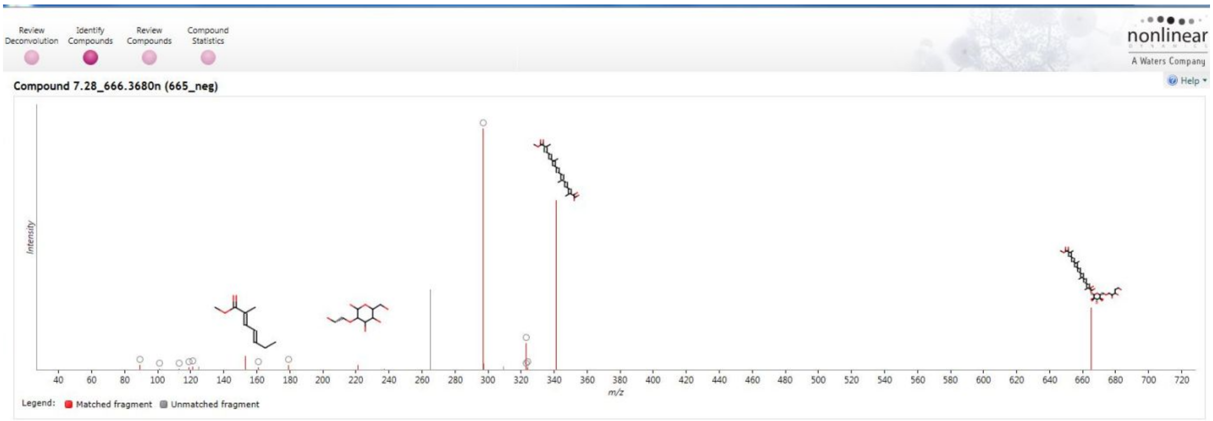

Fig. s23. Fragmentation spectrum of compound 17.

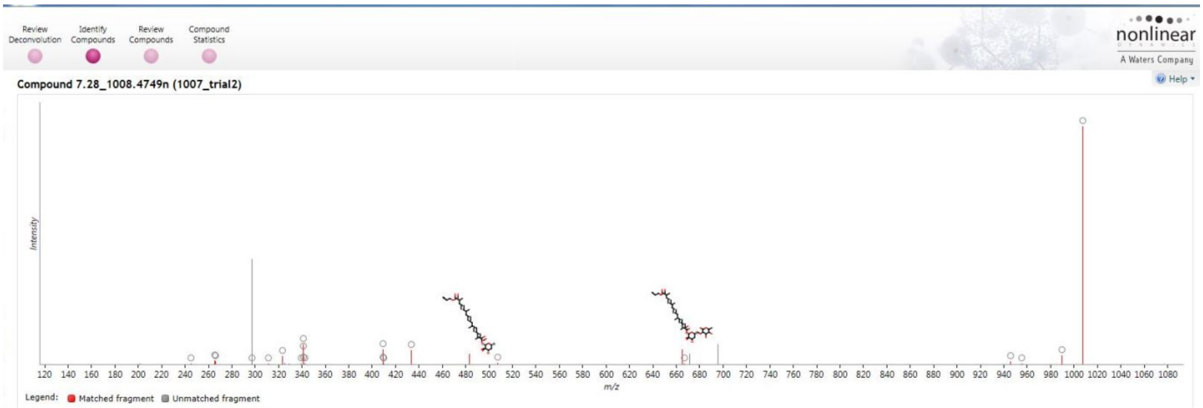

Fig. s24. Fragmentation spectrum of compound 18.

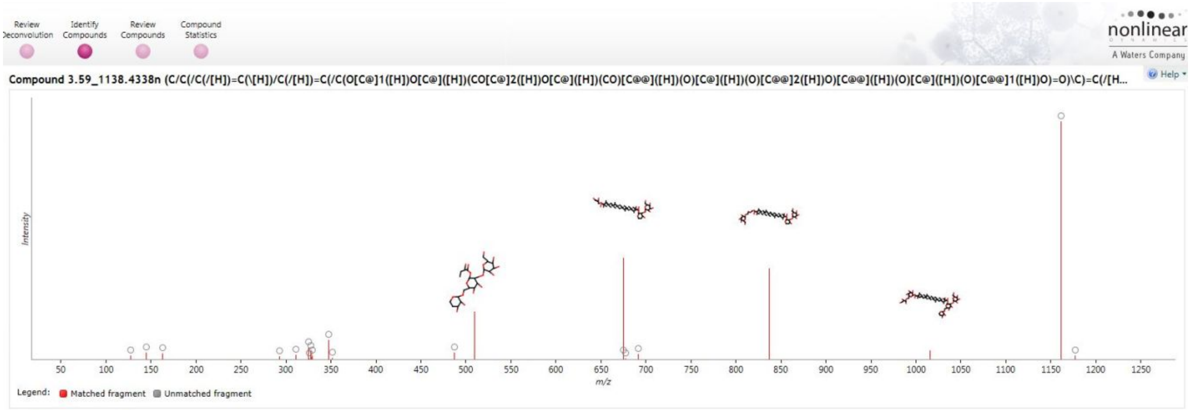

Fig. s25. Fragmentation spectrum of compound 19.

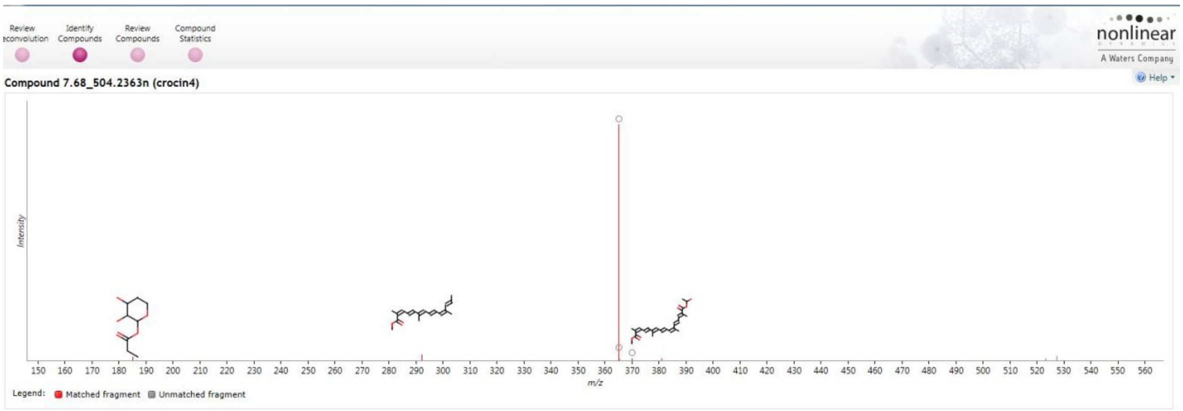

Fig. s26. Fragmentation spectrum of compound 20.

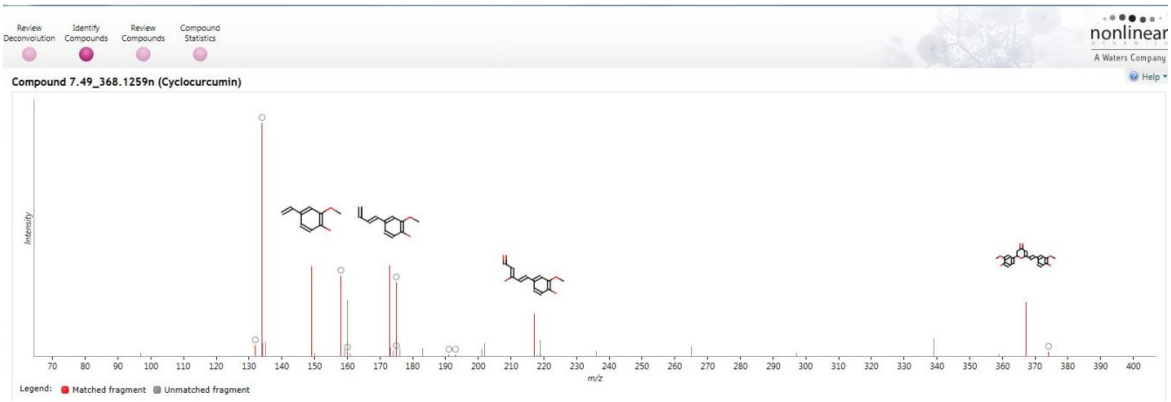

Fig. s27. Fragmentation spectrum of compound 21.

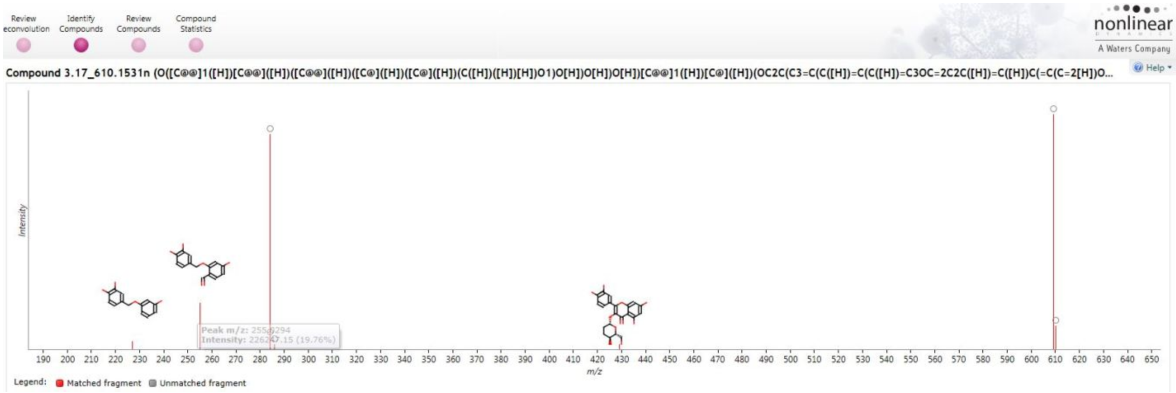

Fig. s28. Fragmentation spectrum of compound 22.

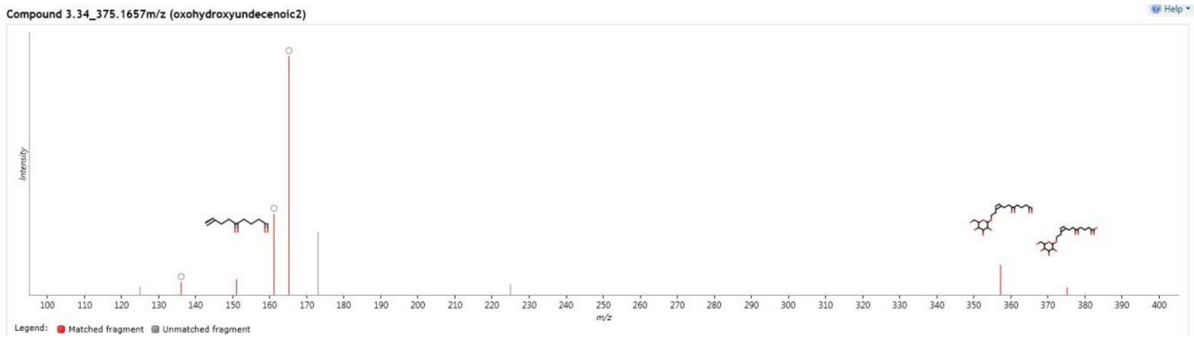

Fig. s29. Fragmentation spectrum of compound 23.

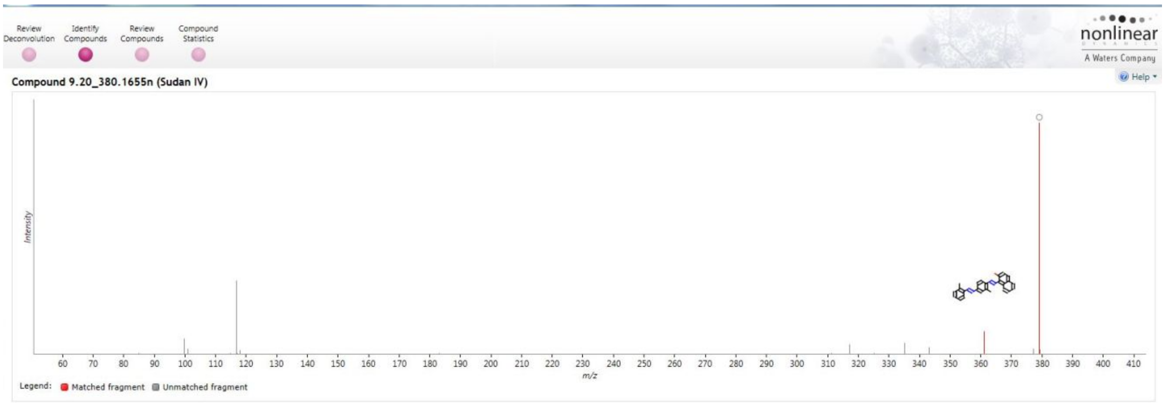

Fig. s30. Fragmentation spectrum of compound 24.

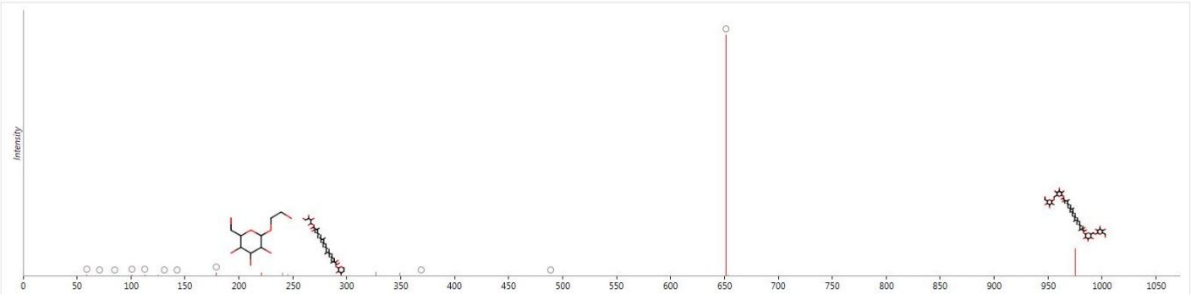

Fig. s31. Fragmentation spectrum of compound 25.

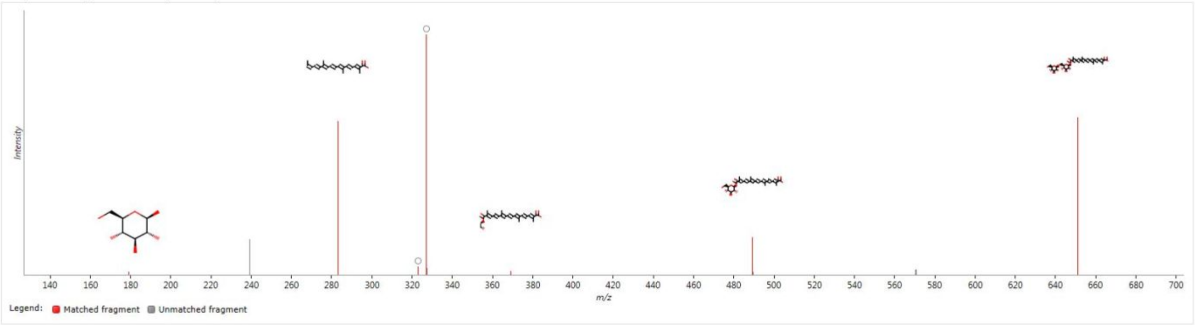

Fig. s32. Fragmentation spectrum of compound 26.

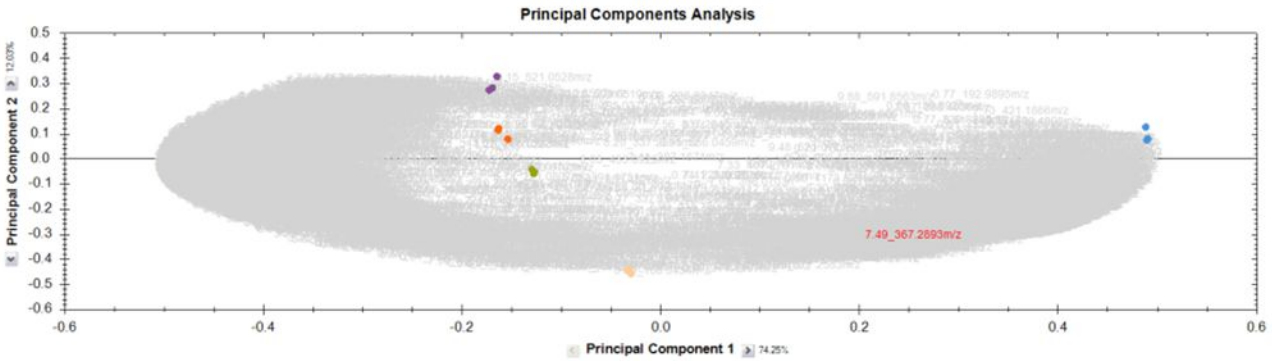

Fig. s33. Score plot of the adulteration of quality control (purple) with curcuma. Addition of 10% (red), 25% (green), 50% (yellow) show a linear trend along PC2. Blue dots represent curcuma.

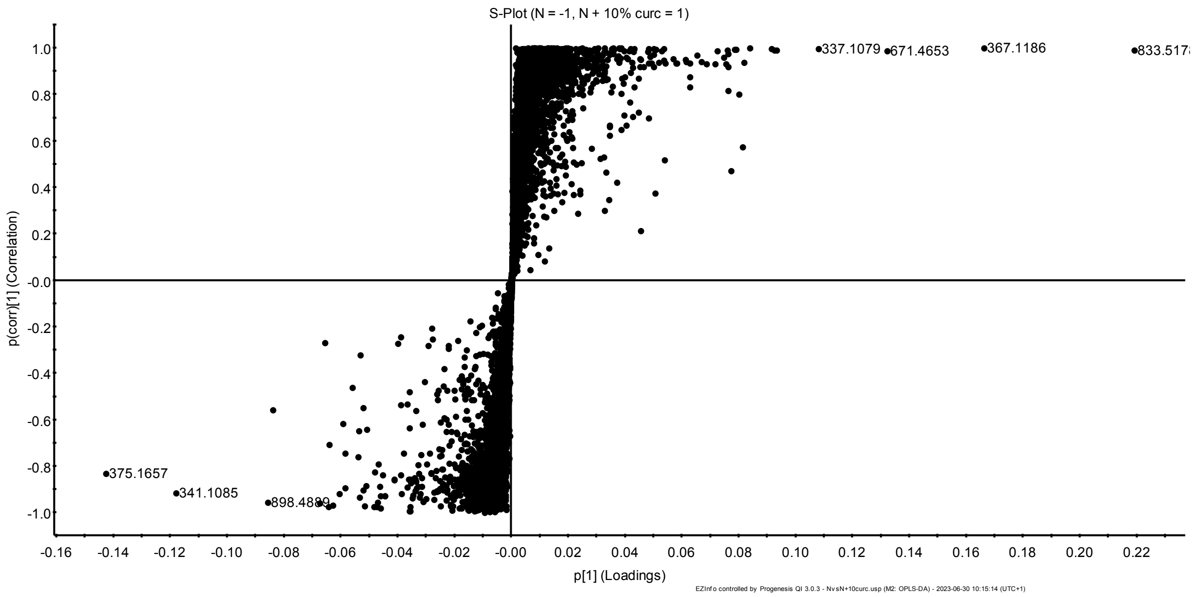

Fig. s34. S-Plot of the adulteration of sample N with 10% turmeric.

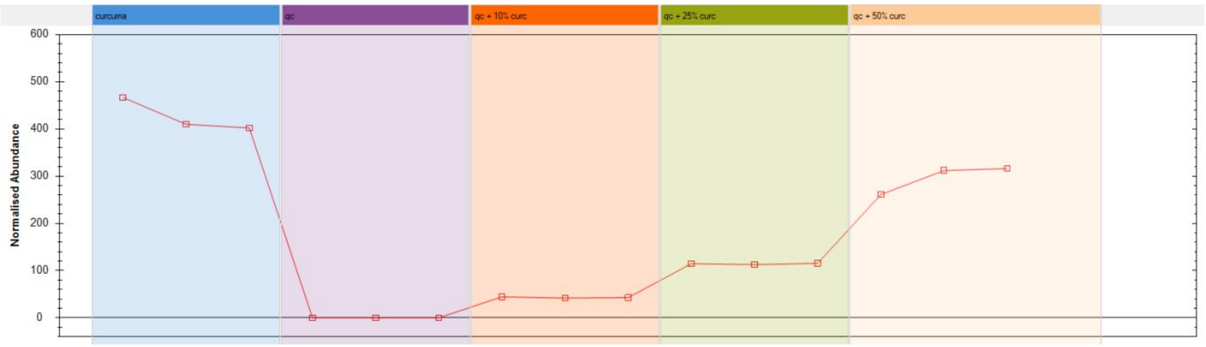

Fig. s35. Trend plot of the m/z 367.1182 (cyclocurcumin, compound 21) in the adulteration of the QC sample.

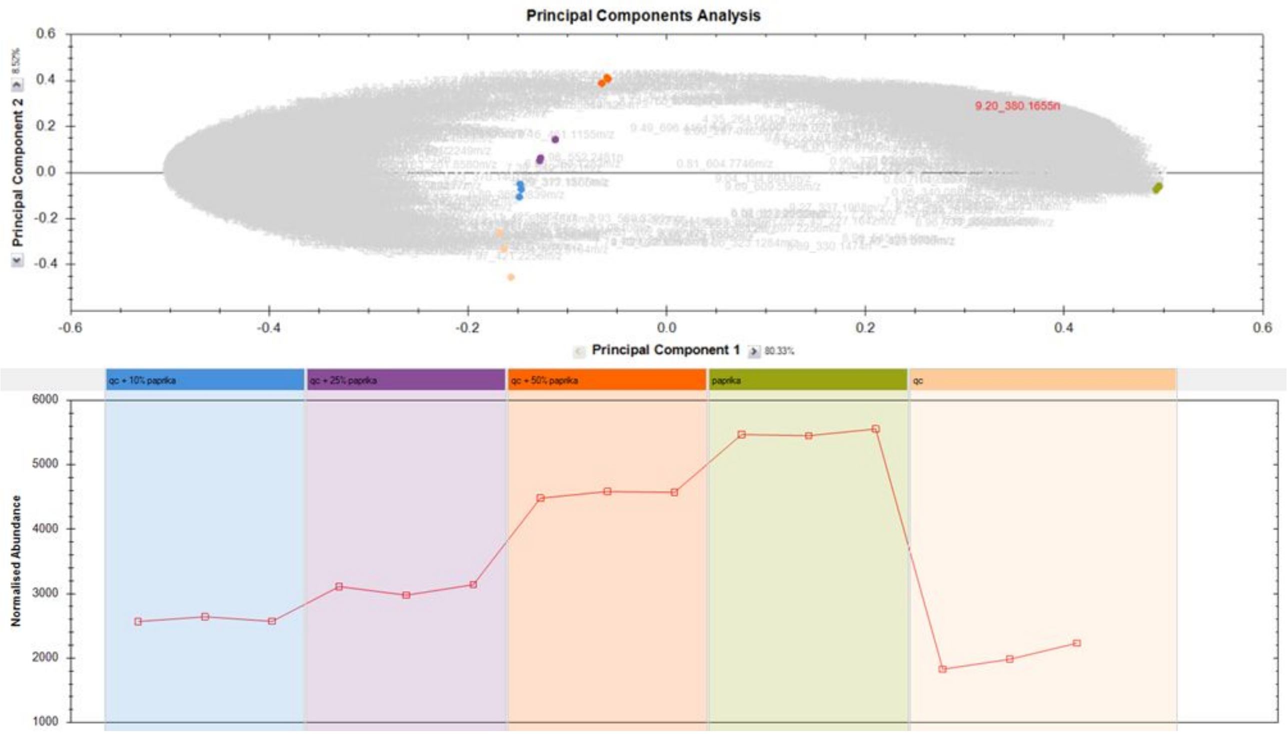

Fig. s36. Score plot and chemometric trend plot of m/z 379.1581 in the QC adulterated with paprika experiment.

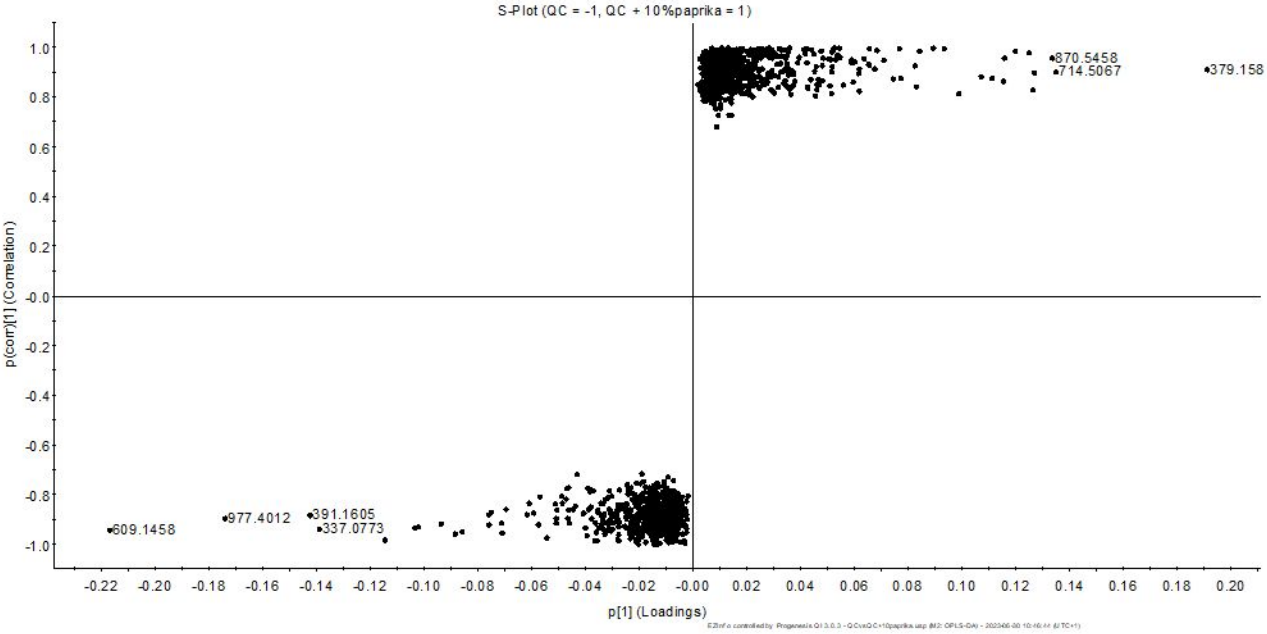

Fig. s37. S-Plot between the quality control (QC) and QC added with 10% of paprika.

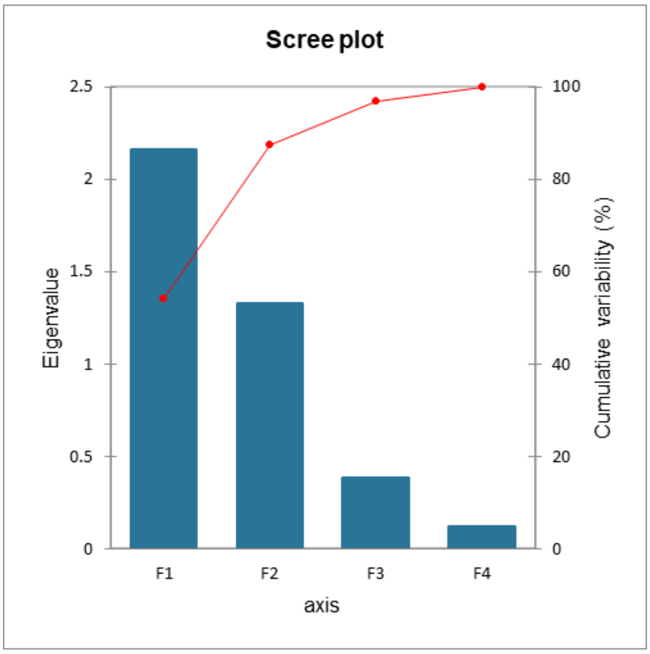

Fig. s38. Scree plot of the PCA reported in Fig. 6.

Table S1. Instrument parameters of the Vion IMS qTOF

**General parameters**	
Analyzer Mode	Sensitivity
**ESI LockSpray**	**Set**
Capillary [kV]	1.50
Sampling Cone [V]	30
Source Offset [V]	80
Source Temperature [°C]	150
Desolvation Temperature [°C]	450
Cone Gas [L/hour]	50
Desolvation Gas [L/hour]	850
Reference Capillary [kV]	1.50
**Instrument**	
Collision Energy [V]	6
Ion Guide Gradient [V]	0.5
Aperture 2 [V]	0.0

LM Resolution (low)	4.7
HM Resolution (high)	15.00
Pre-filter [V]	10
Ion Energy [V]	1.2
Detector Voltage [V]	2700
Average Single Ion Intensity	6.2
Gate Delay [ms]	0
**StepWave**	
SW 1 Offset [V]	20
SW 2 Offset [V]	30
SW 1 Velocity [m/s]	300
SW 1 Pulse Height [V]	15.0
SW 2 Velocity [m/s]	300
SW 2 Pulse Height [V]	15.0
**System2**	
pDRE Attenuate	Off
pDRE Transmission [%]	1.0
pDRE HD Attenuate	Off
pDRE HD Transmission [%]	10.0
**MS Profile**	
Quadrupole Options	Automatic Profile
MS/MSMS	MS
Set Mass	0.00
m/z	1.25 Ma
	0.17Mb
Dwell Time [%Scan Time]	25
Ramp Time [%Scan Time]	75
**RF**	
StepWave RF [V]	250
Trap/IMS RF [V]	250

Ion Guide RF Offset [V]	200
Ion Guide RF Gain	5
Cell 1 RF [V]	300
Cell2 RF Offset [V]	150
Cell2 RF Gain	2
MS/MS Ramp Mode	Automatic
MS/MS Ramp Initial	50
MS/MS Ramp Final	275
**MSe Experiment**	
Mode	High Definition MSe
**Scan Settings**	
Low Mass [m/z]	50
High mass [m/z]	1000
Scan Time [s]	1.000
**Collision energy**	
Low Energy [eV]	6.00
High Energy Ramp [eV]	20.00
to [eV]	40.00
**Lock Correction**	
Automatic Lock Correction Interval [min]	0.5
**Binary Solvent Manager**	
Solvents: A1	H_2O + 0.1% FA
Solvents: B1	ACN + 0.1% FA
Gradient Start	At injection
Seal wash	Enable seal wash
Interval [min]	5.0
**Sample Manager FTN**	
**Solvents**	
Wash Solvent	ACN + 0.1% FA

Pre-inject [s]	0
Post-inject [s]	5
Purge Solvent	H_2O
**Temperatures**	
Sample Temperature [°C]	10.0
Column Temperature [°C]	50.0

Table s2. Results of total crocin, total phenolic and antioxidant activity with the DPPH kinetic method are reported for all the samples with % standard deviation.

Sample	Total Crocin Content (mg mL^{-1})	sd%	Total Phenolic Content mg mL^{-1}	sd%	Antioxidant Activity k_1 ($M^{-1}s^{-1}$)	sd%	Antioxidant Activity k_2 ($M^{-1}s^{-1}$)	sd%
A	1.13	8.48	0.145	3.8	3310	3.1	122	1.1
B	0.66	5.07	0.132	0.9	5390	7.1	132	4.7
C	0.59	1.14	0.123	2.2	2430	14.8	114	6.2
D	0.70	2.13	0.149	0.5	2680	15.4	108	3.4
E	0.93	8.44	0.141	4.8	145000	14.8	237	5.8
F	1.00	2.47	0.144	2.9	13000	11.1	194	9.0
G	1.02	0.51	0.155	2.6	8400	5.5	142	9.2
H	0.53	5.71	0.129	4.5	3900	15.5	136	14.3
I	0.82	12.48	0.166	0.1	3170	10.0	112	6.0
J	0.83	3.06	0.154	1.2	2740	18.6	113	6.4
K	0.82	8.89	0.154	2.6	3040	12.6	108	6.7
L	0.88	2.32	0.130	2.2	5420	9.5	184	9.4
M	0.71	12.54	0.154	0.3	5760	6.2	131	11.9
N	0.61	5.33	0.118	0.5	4170	16.3	149	8.7
